# Photochemical modification of two fluorene-based molecules with DNA intercalating and anti-methicillin resistant *Staphylococcus aureus* activity

**DOI:** 10.1016/j.jbc.2026.113133

**Published:** 2026-05-08

**Authors:** Avery Gaudreau, Matthew D. Beckner, Chenfangfei Shen, Vincent Du, Ronald S. Flannagan, Varsha Balaji, Evangelos Papalambropoulos, Omar M. El-Halfawy, Elizabeth R. Gillies, David E. Heinrichs

**Affiliations:** 1 Department of Microbiology and Immunology, The University of Western Ontario, London, Ontario, Canada; 2 Department of Chemistry, The University of Western Ontario, London, Ontario, Canada; 3 Department of Chemistry and Biochemistry, University of Regina, Regina, Saskatchewan, Canada; 4 Department of Chemical and Biochemical Engineering, The University of Western Ontario, London, Ontario, Canada

**Keywords:** MRSA, *S. aureus*, antibiotic, MOA, infection model, DNA intercalation, photoconversion

## Abstract

*Staphylococcus aureus* is a leading cause of skin and soft tissue infections, endocarditis, and bloodstream infections worldwide. The emergence of methicillin-resistant *S. aureus* (MRSA) and growing resistance to last-resort antibiotics like vancomycin have created an urgent need for new antimicrobials with distinct mechanisms of action. In this study, we characterize DB10, a planar, fluorene-based compound identified in a high-throughput screen for MRSA growth inhibitors. Upon UVA exposure, DB10 undergoes photoconversion from a red-colored form (DB10-R) to a yellow-colored form (DB10-Y). In comparison with DB10-R, DB10-Y exhibits reduced hydrophobicity, lower cytotoxicity, and modestly improved minimum inhibitory concentrations toward several Gram-positive bacteria. DB10-Y intercalates into DNA and induces double-strand breaks within bacterial cells, and resistance emerged only at low levels after prolonged serial passaging. To optimize this scaffold, we screened a panel of fluorene analogs and identified the photoconverting analog DB33, which in its yellow form (DB33-Y) is nontoxic and retained DNA intercalating activity. DB33-Y was effective against intracellular *S. aureus* in macrophages and endothelial cells and significantly reduced bacterial burden and lesion size in a murine skin infection model. DB10-Y and DB33-Y both also suppressed expression of α-hemolysin at sub-minimum inhibitory concentrations, indicating an additional antivirulence effect. Together, these findings highlight the therapeutic potential of fluorene-based DNA intercalators as a new class of antimicrobial and antivirulence agents against MRSA.

*Staphylococcus aureus* is an opportunistic pathogen of significant concern that causes a range of infections, varying from superficial skin and soft tissue infections to severe conditions such as pneumonia, osteomyelitis, and sepsis (1). As one of the leading causes of bacteremia and endocarditis worldwide, *S. aureus* accounts for a significant proportion of nosocomial infections (1). The substantial clinical burden of *S. aureus* is further complicated by its remarkable ability to display resistance to virtually every antibiotic introduced into clinical use (2). In particular, methicillin-resistant *S. aureus* (MRSA) remains a leading cause of morbidity and mortality in both healthcare and community settings, and resistance has increasingly been observed against glycopeptides, lipopeptides, and linezolid, which are among the few remaining treatment options (3, 4). This persistent rise in resistance highlights an urgent need for new antimicrobial agents that target bacterial vulnerabilities beyond the scope of traditional drug targets such as cell wall synthesis, protein translation, and DNA replication (5).

A promising class of antimicrobial agents includes compounds that target bacterial DNA through intercalation and strand damage (6, 7, 8). DNA intercalators act by inserting planar, aromatic moieties—such as those found in naphthoquinones, anthracyclines, and fluorenes—between base pairs of the DNA helix, distorting its structure and disrupting critical processes like replication, transcription, and repair (9, 10). Several DNA intercalators, including well-studied drugs like doxorubicin, have demonstrated antimicrobial activity in addition to their anticancer properties (11, 12). These compounds often exert a multifaceted mechanism of action, not only damaging DNA directly but also generating reactive oxygen species (ROS), disrupting gyrase or topoisomerase activity, or triggering broader stress responses within bacterial cells (13, 14). Other intercalators, including those with naphthoquinone, indole, and fluorene moieties, have shown activity against MRSA, highlighting the potential of this compound class for use as anti-staphylococcals (14, 15, 16, 17, 18).

Beyond their activity against planktonic cells, many DNA-targeting agents also exhibit efficacy against biofilms, likely due to their ability to bind and disrupt extracellular DNA, a critical structural component of the biofilm matrix (10, 19). This is particularly relevant for pathogens like *S. aureus* that form resilient biofilms that contribute to chronic infections and treatment failure (20). Furthermore, these compounds can promote the curing of resistance plasmids, either by directly damaging plasmid DNA or by interfering with replication and segregation, thereby resensitizing bacteria to antimicrobials and limiting the spread of resistance (21). Collectively, these properties highlight DNA-intercalating compounds as promising candidates for antimicrobial development.

However, despite their potential as antimicrobials, the use of DNA-targeting agents is often limited by their toxicity to host cells. Many of these compounds lack sufficient specificity for bacterial DNA and can induce genotoxic effects in mammalian cells, limiting their clinical utility (21, 22). Moreover, DNA intercalating agents are often hydrophobic, a property that facilitates their insertion between DNA bases (23). However, this hydrophobicity also limits clinical viability due to poor solubility and a tendency to precipitate out of aqueous solutions (24). Additionally, DNA intercalation may promote resistance in the long term. Specifically, DNA damage can activate the bacterial SOS response, a global stress pathway that enhances mutagenesis through error-prone DNA polymerases, potentially accelerating the emergence of resistant phenotypes, not only to the DNA-targeting agent itself but also to unrelated antibiotics (25, 26). Nevertheless, the benefits of DNA intercalators can outweigh these limitations, many of which can be addressed through rational structural modifications following the identification of a suitable lead compound.

Here, we define the mechanism of action and spectrum of activity of a planar, red-colored, fluorene-containing compound named DB10, identified from a high-throughput screen for inhibitors active against MRSA. Interestingly, upon UVA exposure, DB10 photoconverted from a red-colored compound (DB10-R) to a yellow-colored compound (DB10-Y). The corresponding structural change modestly lowered the minimum inhibitory concentration (MIC) across most bacterial isolates tested, but significantly reduced the hydrophobicity and toxicity of the compound. Given these improved properties, DB10-Y was pursued as the lead compound. As predicted based on its fluorene core, DB10-Y intercalated into DNA. Attempts to generate resistance to B10-Y resulted in only low-level resistance after 46 days of serial passaging in the presence of sub-MIC compound. To examine key structural features of this molecule class, a series of fluorene analogs were obtained and, of these, only one, DB33, retained strong antimicrobial activity. Like DB10, DB33 intercalated into DNA but, notably, toxicity was not observed. In addition, DB33 was effective against intracellular *S. aureus* reservoirs and reduced bacterial burden and lesion size in a murine skin infection model. Furthermore, at sub-MIC concentrations we observed a decrease in production of *S. aureus* α-hemolysin (encoded by the *hla* gene), indicating an antivirulence effect. Together, these findings highlight the therapeutic potential of the DB10 scaffold and underscore the broader utility of planar aromatic intercalators as a class of antimicrobials.

## Results

### High-throughput screen identifies a photoconvertible inhibitor of MRSA

**Figure 1:**
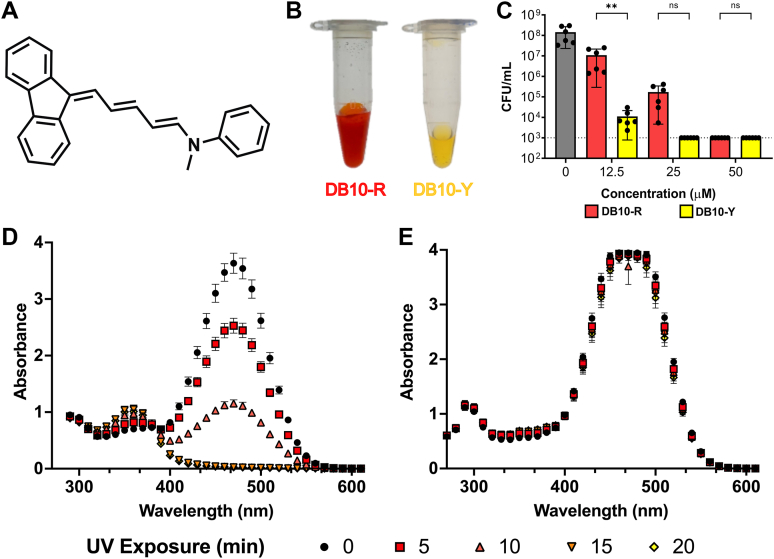
**DB10 is a photoconvertible inhibitor of *S. aureus* USA300 LAC.***A*, chemical structure of DB10. In (*B*) the image depicts aliquots of DB10 before (DB10-R) and after (DB10-Y) photoconversion. *C*, killing of *S. aureus* following 24 h exposure to DB10-R or DB10-Y at the indicated concentrations. Bacteria were normalized to OD_600_ = 1 in PBS, treated with compound or vehicle, and incubated at 37 °C for 24 h before serial dilution and plating on TSA to enumerate CFU. Data are shown as the mean ± SD from at least three biological replicates. ∗∗*p* ≤ 0.01 using a one-way ANOVA with Dunnet’s multiple comparison. *D*, absorbance spectra of DB10-R as it is converted to DB10-Y during long wave UV (UVA, 365 nm) or (*E*) the lack of conversion after short wave UV (UVB, 255 nm) exposure. Absorbance was read every 5 min and data are shown as the mean ± SD from at least three independent experiments. CFU, colony-forming unit.

A high-throughput screen of the Maybridge library of bioactive compounds was previously conducted (27, 28) to identify potent inhibitors of *S. aureus* USA300 LAC, a predominant community-acquired MRSA clone in North America. This initial screen, performed in tryptic soy broth (TSB), yielded 936 potential inhibitors, though early efforts focused exclusively on compounds suspected to disrupt metal ion homeostasis (28). More recently, we revisited this set of bioactives to identify additional promising scaffolds that showed anti-staphylococcal activity. During this analysis, we observed that roughly 11% of the 936 hits shared a common feature in that they possessed planar, aromatic structures, chemical motifs either known to, or with the possibility to, intercalate into DNA. DNA intercalating agents may disrupt essential bacterial processes such as replication and transcription, making them attractive candidates for antimicrobial development despite potential concerns about genotoxicity (21, 22). However, when this set of 936 initial hits were tested in a Chelex-100–treated, chemically defined medium containing glucose, only three of these planar compounds retained their activity against USA300 LAC. The Chelex treatment removes divalent metal ions, creating a metal-depleted, host-relevant environment where some metal-dependent compounds may lose efficacy, helping to identify inhibitors with robust, metal-independent activity (28, 29). The three active compounds included one with an indole moiety, one with a phenanthroline, and one with a fluorene. Given the relative prevalence of fluorene-containing compounds (∼10%) among the subgroup of hits containing a planar aromatic moiety, and the robust activity of this compound under metal-depleted conditions, we selected it for further study and designated it DB10 (Fig. 1*A* for structure).

Interestingly, during our initial experiments using DB10, we observed a gradual color change from red (DB10-R) to yellow (DB10-Y) after the compound was reconstituted and exposed to glass-filtered sunlight over a period of 3 to 5 days (Fig. 1*B*). This observation suggested that DB10 may undergo photoconversion, a phenomenon in which light exposure induces a chemical change in a compound and also potentially alters its biological activity or stability. Indeed, photoconversion of DB10-R to DB10-Y altered the bactericidal activity of these compounds against *S. aureus*. While both forms exhibit similar efficacy at 50 μM, DB10-Y consistently results in lower colony-forming unit (CFU)/ml counts at 12.5 and 25 μM, suggesting that the yellow form is more antibacterial (Fig. 1*C*). Photoconversion also improved the solubility of the compound and, while DB10-R precipitated from aqueous solution at concentrations above 300 μM, DB10-Y remained soluble at concentrations up to 1 mM but precipitated out of solution at concentrations >1 mM.

Next, we sought to investigate the mechanism behind this suspected photoconversion. To this end DB10 was subjected to either longwave (UVA) or shortwave (UVB) ultraviolet light, and the resulting color change was monitored over time by absorbance (Fig. 1, *D* and*E*). Upon UVA exposure, a time-dependent decrease in absorbance at ∼470 nm was observed, corresponding to the disappearance of the red-colored species (DB10-R). Concurrently, the absorbance around the ∼370 nm peak increased, consistent with the formation of the yellow photoproduct (DB10-Y). Complete photoconversion of DB10-R occurred within 15 min of UVA exposure. In contrast, when the same concentration of DB10-R was exposed to UVB, no appreciable color change was observed, and the absorbance peak at ∼470 nm remained stable and did not decrease. This strongly suggested that UVB exposure was insufficient to induce any chemical transformation of DB10, at least under the tested conditions, and that the photoconversion of DB10-R to DB10-Y was driven by UVA. To confirm the structure of DB10-R and assess the structural basis of this conversion, ^1^H NMR spectroscopic analyses was performed (Fig. S1). Complete photoconversion was achieved after 44 h of UV exposure at 460 nm, as indicated by the complete disappearance of peak “b”, corresponding to a characteristic proton on the conjugated alkene system. The longer light exposure required for the NMR sample compared to the UV-visible spectroscopy sample can be attributed to the higher concentration, which reduces light penetration. New peaks arose at 8.6 and 4.7 ppm, which may arise from oxidative cleavage of the alkenes, resulting in an aldehyde and hydrated aldehyde product as characterized previously for conjugated alkene systems such as astaxanthin and cyanine dyes upon irradiation in the presence of oxygen (30, 31).

### Photoconversion enhances activity of DB10 against Gram-positive bacteria

To further assess the spectrum of activity and bacterial response to both DB10-R and DB10-Y, we determined the MICs of each compound against various *S. aureus* strains, other *Staphylococcus* species, and non-staphylococcal bacteria. In Mueller-Hinton broth (MHB), both DB10-R and DB10-Y inhibited most *Staphylococcus* isolates tested (Table 1), as well as other Gram-positive species at concentrations ≤ 50 μM. However, at the highest concentration tested (100 μM), growth persisted for *Staphylococcus cohnii*, *Staphylococcus warneri*, *B. subtilis*, and *Staphylococcus pyogenes* in the presence of DB10-R. Neither form of the compound at the concentrations tested here showed activity against the Gram-negative species evaluated (Table 1). Although not statistically significant for most isolates, and consistent with CFU counts, DB10-Y consistently exhibited slightly lower MIC values than DB10-R, suggesting a modest increase in potency following photoconversion.

**Table 1:** DB10-R and DB10-Y are inhibitors of Gram-positive bacteria

Bacterial isolate	DB10-R (μM)	DB10-Y (μM)
*S. aureus* USA100	50	25
*S. aureus* USA200	50	25
*S. aureus* USA300	50	25
*S. aureus* USA400	50	50
*S. aureus* USA600	100	100
*S. capitis*	50	25
*S. epidermidis*	50	25
*S. chromogenes*	50	25
*S. lugdunensis*	50	50
*S. cohnii*	>100	>100
*S. warneri*	>100	50
*B. subtilis*	>100	100
*S. pyogenes*	100	50
*S. agalactiae*	50	50
*E. faecalis*	25	25
*P. aeruginosa*	>100	>100
*E. coli*	>100	>100

MICs of select *S. aureus* strains, Staphylococcal spp. and non-staphylococcal spp. The concentration of DB10-R or DB10-Y that completely inhibited bacterial growth was determined to be the MIC. At least three biological replicates for each isolate and form of DB10 were conducted to determine the MICs.

### *S. aureus* alters its transcriptome upon exposure to DB10-R and DB10-Y

**Figure 2:**
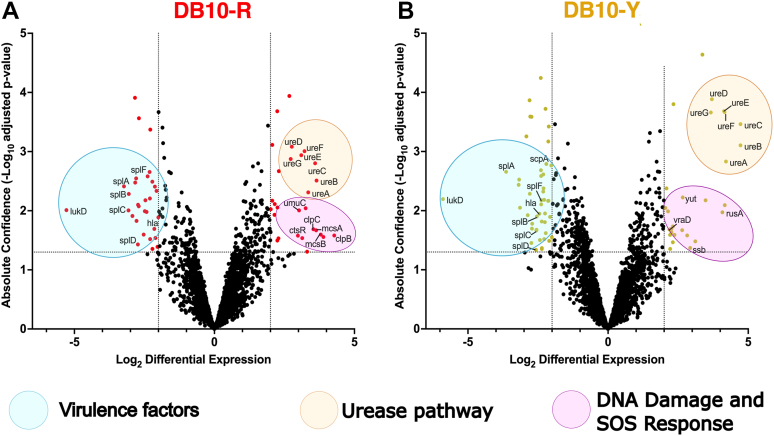
**DB10-R and DB10-Y elicit similar transcriptional responses in *S. aureus*.** Volcano plots of RNA-seq analysis of *S. aureus* USA300 LAC after exposure to 50 μM (*i.e.* sub-MIC in TSB) of either (*A*) DB10-R or (*B*) DB10-Y for 1 h during early exponential phase growth. Results are from three independent experiments. Differentially expressed genes (absolute confidence >2, |log2 differential expression| >1.3) are colored in *red dots* for DB10-R and *yellow dots* for DB10-Y. Differentially expressed pathway groups are encircled in *blue* (virulence factors), *orange* (urease pathway), or *purple* (DNA damage and SOS response). Detailed RNAseq data can be found in Table S4 (RNAseq for *S. aureus* exposure to DB10-R) and Table S5 (RNAseq for *S. aureus* exposure to DB10-Y). MIC, minimum inhibitory concentration; SOS, a cellular response to DNA damage; TSB, tryptic soy broth.

To understand how *S. aureus* responds to each compound and whether DB10-R and DB10-Y evoked conserved transcriptional responses, we performed RNA-seq on *S. aureus* USA300 LAC cultures exposed to sub-MIC concentrations of each compound for 1 h during early exponential growth phase. Although MICs in Table 1 were determined in Mueller-Hinton broth (MHB), the standard medium for antimicrobial testing, *S. aureus* is more typically grown in TSB, which is also the conventional medium for transcriptomic analyses. Notably, the MICs for both DB10-R and DB10-Y in TSB are approximately 100 μM. Untreated cultures were included as controls to establish baseline gene expression. A total of 59 and 71 differentially expressed genes were identified following exposure to DB10-R (Fig. 2*A*) and DB10-Y (Fig. 2*B*), respectively.

DB10-R and DB10-Y both induced expression of genes associated with stress and DNA damage responses. Additionally, DB10-R treatment led to significant upregulation of *clpB* and *clpC*, which encode Clp family proteases involved in refolding or degrading misfolded proteins under stress conditions (32). DB10-R also increased expression of *umuC*, *ctsR*, *mcsA*, and *mcsB*, while DB10-Y upregulated *rusA* and *ssb*. In *S. aureus*, *umuC* is part of the SOS response and encodes a DNA polymerase that bypasses damaged DNA (33). *ctsR* regulates heat shock genes, while *mcsA* and *mcsB* are involved in controlling Clp-dependent proteostasis under stress (34). *rusA*, although better characterized in *Escherichia coli,* likely functions similarly in resolving Holliday junctions (35), and *ssb* stabilizes single-stranded DNA during replication and repair (36). Collectively, our data suggest that both forms activate cellular pathways involved in genome maintenance and repair. Interestingly, both treatments also resulted in upregulation of the *ureABCDEF* operon, which encodes urease and associated accessory proteins (37, 38). Urease catalyzes the hydrolysis of urea into ammonia and carbon dioxide and plays a known role in neutralizing intracellular pH during acid stress (38). Upregulation of this operon suggests a broader cellular response to intracellular acidification, metal dysregulation, or other stress-related cues triggered by DB10 exposure. Notably, both forms of DB10 significantly downregulated numerous virulence factors, including the *spl* serine protease-like operon, *hla* (encoding α-hemolysin), and *lukD* (a component of the leukocidin cytotoxin) (Fig. 2).

Collectively, these data demonstrate that DB10-Y elicits similar transcriptional changes on *S. aureus* as DB10-R. Based on that, and our findings that DB10-Y also has improved solubility and modestly enhanced potency (preceding section), we therefore selected DB10-Y for further studies.

### DB10-Y intercalates into DNA and causes intracellular dsDNA breaks

As mentioned previously, DB10-Y is a planar, aromatic compound with a core fluorene moiety. Considering other compounds with this moiety are known to either intercalate or interact with DNA in some way (39, 40), along with the induction of the DNA damage response as observed in our RNA-seq after DB10-Y exposure, we speculated that the main mechanism of action of this inhibitor would involve DNA binding in some manner.

**Figure 3:**
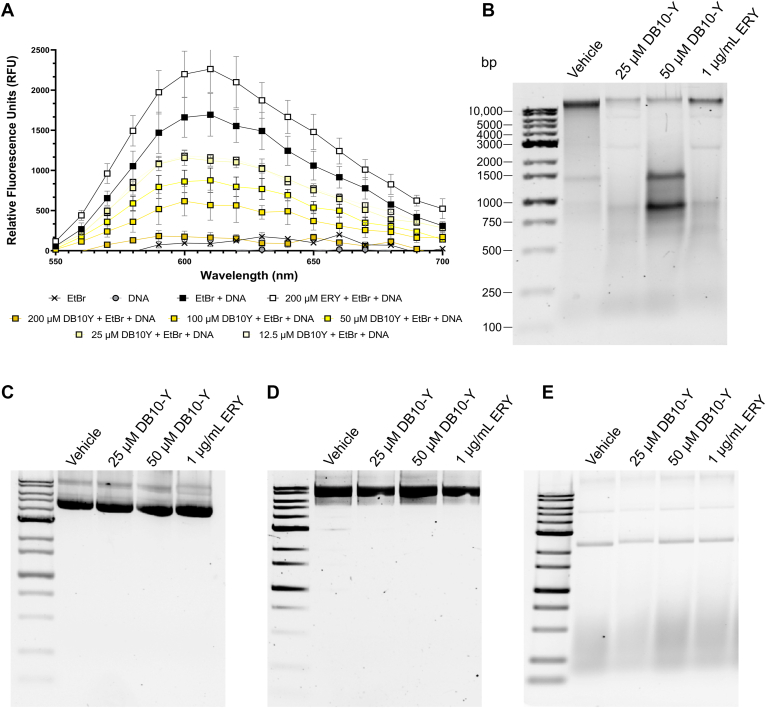
**DB10-Y competitively intercalates into DNA and causes intracellular DNA damage.***A*, DB10-Y displaces EtBr from DNA. EtBr, DNA, and various concentrations of DB10-Y, or the control erythromycin were incubated together for 30 min in the dark before the fluorescence spectra of EtBr was read (excitation 525 nm). DB10-Y, EtBr, and DNA alone showed no intrinsic fluorescence, and ERY, a nonintercalating control, had no effect on EtBr fluorescence. Data are shown as the mean ± SD from at least three biological replicates. *B*, *C*, and *D*, representative agarose gel images. *B*, gDNA was isolated from *S. aureus* USA300 LAC subcultured overnight in the presence of DB10-Y or ERY. A total of 500 ng/μl was treated with TE buffer to induce unwinding before DNA was loaded onto the gel. A total of 100 ng/μl of empty pALC2073 plasmid isolated from USA300 LAC was (*C*) left uncut or (*D*) linearized with *Sac*I prior to 1 h treatment with DB10-Y or ERY after isolation. *E*, gDNA (50 ng/μl) isolated from untreated USA300 LAC was treated with DB10-Y or ERY for 1 h prior to electrophoresis. All samples were resolved on 0.8% agarose TBE gels at 100 V. Accumulation of shorter DNA fragments is indicative of double-strand breaks (DSBs). Ladder for all agarose gels (*B*-*E*) was a 1 kB Plus DNA ladder (FroggaBio), with band sizes indicated in 3B. Images are representative of three independent experiments. ERY, erythromycin; EtBr, ethidium bromide; TE, tris-EDTA.

To evaluate the DNA intercalative properties of DB10-Y, we performed a competitive intercalation assay using ethidium bromide (EtBr), a well-established DNA intercalator that fluoresces upon binding to DNA (41). In this assay, EtBr is allowed to intercalate into DNA, and the subsequent addition of compounds that compete for intercalation sites displace EtBr, resulting in a measurable reduction in fluorescence (15). As expected, increasing concentrations of DB10-Y resulted in a dose-dependent decrease in EtBr fluorescence, indicating that DB10-Y competitively displaces EtBr (Fig. 3*A*). Notably, DB10-Y alone, whether in the presence or absence of DNA, did not emit detectable fluorescence under the assay conditions, confirming that the observed signal changes were attributable solely to EtBr displacement. To confirm the specificity of this effect, we included erythromycin (ERY), a nonintercalative control compound. ERY did not alter EtBr-DNA fluorescence, reinforcing our conclusion that the fluorescence decrease observed with DB10-Y was due to its specific displacement of EtBr from DNA. Furthermore, this intercalative ability was not specific to genomic DNA (gDNA) isolated from *S. aureus*. Eukaryotic gDNA from RAW 264.7 macrophages exhibited similar levels of intercalation compared to *S. aureus* gDNA (Fig. S2).

Given that intercalating agents can induce intracellular DNA damage, we next assessed whether DB10-Y caused DNA strand breaks. gDNA was isolated from cultures exposed to DB10-Y and subjected to electrophoresis under either neutral or alkaline conditions to differentiate between double-strand breaks (DSBs) and single-strand breaks (SSBs), respectively, as described previously (42). Under neutral conditions (pH 7.5), DB10-Y treatment resulted in the accumulation of shorter DNA fragments, consistent with the presence of DSBs (Fig. 3*B*). In contrast, the DNA banding pattern under alkaline conditions was similar to that observed under neutral conditions, indicating no detectable increase in SSBs (Fig. S3*A*). A modest decrease in band intensity was observed for the ERY nonintercalating control relative to the untreated sample, which is consistent with the known effects of translation inhibition on nucleoid organization and DNA compaction (43). When cultures are pretreated prior to DNA isolation, these changes can influence DNA fragmentation and electrophoretic migration, resulting in altered band appearance despite comparable DNA preparations (43).

To determine whether this damage resulted from direct chemical cleavage of DNA, purified substrates were tested under the same conditions used for the *in vitro* intercalation assays. DB10-Y failed to induce strand breaks in uncut plasmid DNA (Fig. 3*C*), *Sac*I-digested plasmid DNA (Fig. 3*D*), or gDNA previously isolated from untreated cells (Fig. 3*E*), despite clear evidence of intercalation (Fig. 3*A*). These results indicate that DNA strand breaks arise indirectly from cellular processes triggered by DB10-Y exposure rather than from direct DNA cleavage.

ROS, including superoxide, have been implicated in mediating DNA damage in response to bactericidal antibiotics (44). To evaluate whether oxidative stress contributes to the DNA damage observed in *S. aureus* cells, we assessed the production of superoxide using the nitroblue tetrazolium (NBT) assay, which detects superoxide through the reduction of NBT to a blue formazan precipitate (45). Treatment of *S. aureus* cells with DB10-Y did not result in detectable ROS production, as no significant superoxide levels were observed (Fig. S3*B*), suggesting that the observed DNA damage is a direct effect of DB10-Y rather than a consequence of oxidative stress.

Despite evidence of DNA intercalation and DSBs, transcriptomic analysis revealed no upregulation of key DNA damage or stress response genes, including *recA* and *rexAB* (Fig. 2*B*). Furthermore, transposon mutants of these genes did not exhibit altered sensitivity to DB10-Y (Fig. S3*C*). Together, these results suggest that while the primary MOA of DB10-Y involves DNA intercalation, additional mechanisms likely contribute to its antibacterial effect.

### Mutations in *clpX* confer low-level resistance to DB10-Y

**Figure 4:**
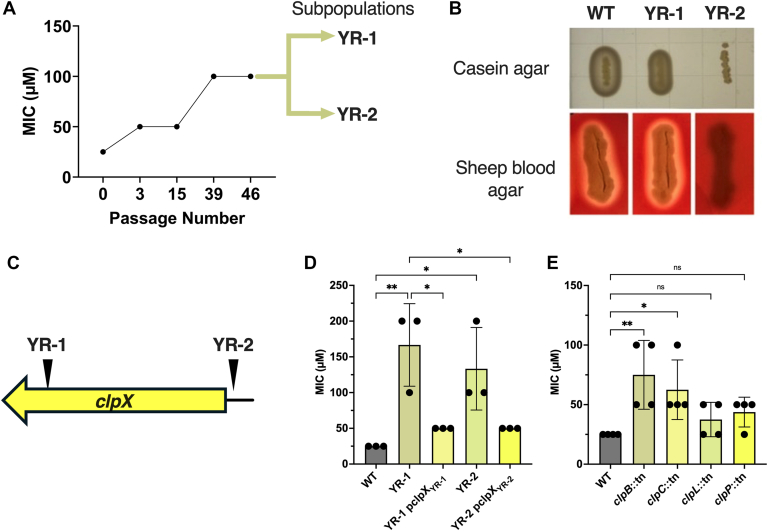
**SNPs in *clpX* confer low-level resistance to DB10-Y.***A*, resistance development timeline of USA300 LAC exposed to sub-MIC (50 μM in TSB). Resistance was checked periodically until an increase in resistance was observed (day 39) at which time additional passaging for 7 days was done to ensure mutation stability. *B*, resistant subpopulations can be distinguished by a SNP in *agrC* in YR-2 resulting in a loss of casein hydrolysis and hemolysis on sheep blood agar. *C*, location of SNPs found in and upstream of *clpX* within the two resistant subpopulations. MICs of (*D*) resistant subpopulations overexpressing mutant *clpX* and (*E*) *clp* system transposon mutants. Data are shown as the mean ± SD of at least three independent experiments. ∗*p* ≤ 0.05, ∗∗*p* ≤ 0.01, using a one-way ANOVA with Dunnett’s multiple comparison. MIC, minimum inhibitory concentration; TSB, tryptic soy broth.

A well-established strategy for investigating the MOA of antimicrobials is the selection of resistant mutants through prolonged exposure. To this end, we sought to evolve resistance to DB10-Y by serially passaging cultures of USA300 LAC in the presence of sub-MIC (50 μM in TSB). After approximately 46 days, we obtained low-level but stable resistance to DB10-Y (Fig. 4*A*). The extended time required to develop resistance may further suggest a pleiotropic mechanism of action that is not solely dependent on DNA intercalation and damage.

Interestingly, the resistant culture consisted of two distinct subpopulations, from which we isolated representative strains YR-1 and YR-2. Subpopulation 2 (YR-2) harbored a SNP in *agrC*, which resulted in a loss of casein hydrolysis and hemolytic activity on blood agar (Fig. 4*B*), allowing it to be readily distinguished from subpopulation 1 (YR-1). Aside from the SNP in *agrC*, both subpopulations were found to contain unique SNPs within the *clpX* locus (Fig. 4*C*), which encodes an ATP-dependent chaperone that interacts with ClpP to facilitate protein degradation and regulate stress responses (46). YR-1 harbored a deletion immediately upstream of the +1 transcriptional start site of *clpX*, potentially affecting promoter function and gene expression. YR-2 contained a frameshift-inducing deletion within codon 400, near the C terminus of *clpX*, leading to a premature stop codon. These findings suggest that alterations in *clpX* function could contribute to low-level resistance to DB10-Y.

To investigate this further, the mutant *clpX* alleles were cloned and overexpressed in either YR-1 or YR-2 backgrounds. Rather than further increasing resistance, overexpression of these alleles restored susceptibility to near WT levels (Fig. 4*D*). To determine whether the increased resistance observed in YR-1 and YR-2, and the subsequent loss of resistance after *clpX* overexpression, was specific to DB10-Y, we compared the MICs of these mutants to WT USA300 LAC across other antibiotics. YR-1 displayed increased susceptibility to ofloxacin and vancomycin, while YR-2 showed increased resistance to ampicillin. In both cases, complementation with the corresponding *clpX* allele reversed the phenotype, returning susceptibility to levels comparable to USA300 LAC (Fig. S4). These data suggest that altered *clpX* expression or activity modulates susceptibility not only to DB10-Y but also to other antibiotics, likely through effects on proteostasis or stress response pathways.

To assess whether disruption of other Clp-associated genes similarly affects susceptibility, we screened transposon insertion mutants for changes in DB10-Y resistance (Fig. 4*E*). *clpB* and *clpC* transposon mutants exhibited increased resistance, while insertions in *clpP* and *clpL* did not alter susceptibility. A *clpX* transposon mutant was not available in the NTML, likely because *clpX* is essential under these conditions, preventing direct comparison. These results further suggest that perturbation of Clp-mediated proteostasis can influence susceptibility to DB10-Y. However, the low level of resistance observed, and the extended time required for emergence of this resistance indicate that these mutations likely represent supplementary, off-target adaptations rather than primary resistance mechanisms.

### Screening of DB10-Y analogs identifies DB33-Y

**Figure 5:**
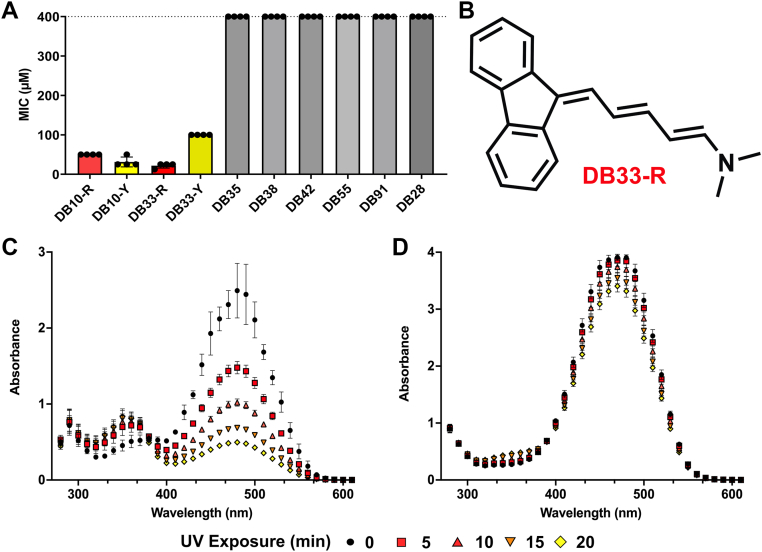
**DB33 is a promising structural analog of DB10.***A*, MICs of DB10-R and DB10-Y with seven selected structural analogs. Analogs with no biological activity are colored in shades of *gray*. *B*, structure of the only biologically active analog, DB33. Photoconversion of DB33-R to DB33-Y using (*C*) long-wave UV and lack of photoconversion after (*D*) short-wave UV exposure. Absorbance was read every 5 min and data are shown as the mean ± SD from at least three replicates. MIC, minimum inhibitory concentration.

Although we initially hypothesized that the central fluorene moiety in DB10-Y was responsible for its ability to intercalate into DNA, previous studies have shown that fluorene alone is not capable of this function (47). To identify structural features critical for antibacterial activity, we tested seven structural analogs of DB10, each retaining the fluorene core but with modified side chains (Fig. S5). Analogs were selected from the Mcule database using the structure similarity search feature. Of these, six analogs exhibited no inhibitory activity against USA300 LAC at concentrations up to 400 μM (Fig. 5*A*). However, one analog, designated DB33 (Fig. 5*B*), displayed potent activity with an MIC of 25 μM, comparable to that of DB10. The structure of DB33-R was confirmed by ^1^H NMR spectroscopy (Fig. S6). DB33, like DB10, undergoes UVA-induced, but not UVB-induced, photoconversion, resulting in a visible color change from red to yellow and a corresponding shift in absorbance peak from 480 nm to 350 nm (Fig. 5, *C* and *D*). Importantly, DB33-R differs from DB10-R only by the presence of a terminal phenyl group, suggesting that broader structural modifications to the fluorene core or conjugated side chain are not well tolerated and result in loss of antimicrobial activity.

Notably, both the red (DB33-R) and yellow (DB33-Y) forms showed similar antibacterial activity to DB10 against a panel of *S. aureus* strains and other species (Table S1). Interestingly, photoconversion of DB33 resulted in a modest increase in MIC (100 μM) against USA300 LAC, opposite to the trend observed for DB10.

To confirm whether DB33-Y shares the same DNA-targeting mechanism as DB10-Y, we once again performed a competitive EtBr displacement assay. As DB33-Y concentration increased, EtBr fluorescence decreased in a dose-dependent manner, indicating that DB33-Y competes with EtBr for DNA binding and intercalates into DNA (Fig. S7*A*). Consistent with the concept of a shared mechanism, *clpX* mutant strains YR-1 and YR-2, originally selected for resistance to DB10-Y, also displayed resistance to DB33-Y, further supporting that the analog DB33-Y shares a common mode of action to DB10-Y (Fig. S7*B*).

We next assessed whether DB10 analogs that did not have appreciable anti-staphylococcal activity (colored in gray in Fig. 5*A*) had the ability to intercalate into DNA by performing the EtBr displacement assay. All exhibited some degree of DNA intercalating activity (Fig. S8); however, EtBr displacement was generally lower than that observed with DB10-Y. Interestingly, DB35 showed slightly greater displacement than DB10-Y, despite lacking antibacterial activity, This observation is consistent with previous reports that fluorene-containing aromatic systems can intercalate DNA to varying extents depending on planarity and conjugation, but that intercalation alone does not necessarily translate to antimicrobial activity (40, 48). In the case of DB35, the absence of antimicrobial effect despite measurable DNA binding suggests that additional properties such as cellular uptake or intracellular stability are likely required for antibacterial efficacy.

### A breakdown product of DB33-R is bioactive

To investigate structural changes associated with the photoconversion of DB33-R and confirm the active component of DB10-Y, we performed ^1^H NMR spectroscopy on DB33 at 9 g/L in DMSO-d_6_. Complete conversion was observed after 31 h of irradiation including bubbling air through the sample to provide oxygen (Figs. S9 and S10). The resulting ^1^H NMR spectrum revealed multiple degradation products, including aldehyde peaks near 10 ppm and methyl peaks between 2.7 to 3.2 ppm, consistent with chain shortening and oxidation. Additionally, the disappearance of the 5.45 ppm alkene proton and downfield shifts indicate cleavage and oxidation of the polyene system. Based on prior studies of polyene dyes and natural products, a number of different chain truncated and oxidized products are possible, accounting for the large number of observed peaks (30, 31, 49, 50, 51).

**Figure 6:**
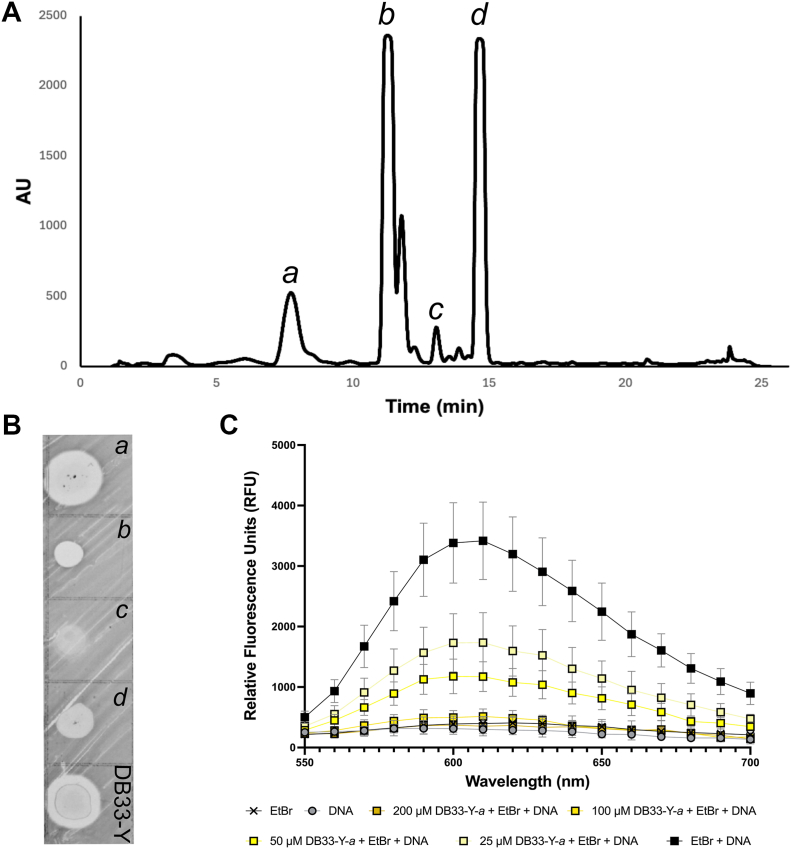
**DB33-R breakdown product *a* is bioactive.***A*, HPLC trace of the photodegradation products making up DB33-Y after conversion from DB33-R. *B*, peaks identified in (*A*) were collected, dried, and resuspended in DMSO. A 1:1 M conversion from starting DB33-Y to each breakdown product was assumed in order to calculate resuspension volumes corresponding to the original DB33-Y stock concentration. Aliquots (3 μl) of each collected peak and the DB33-Y stock were spotted onto MHA plates prestreaked with WT USA300 LAC. Zones of inhibition were imaged after 24 h incubation. *C*, DB33-Y-*a* displaces EtBr from gDNA. Data are shown as the mean ± SD from at least three biological replicates. DMSO, dimethyl sulfoxide.

Although the DB33-Y mixture displayed antimicrobial activity, it remained unclear whether bioactivity arose from synergistic effects among these multiple NMR-identified breakdown products or from a single dominant compound. DB33-Y was therefore separated by HPLC, revealing four major peaks (Fig. 6*A*). Fractions corresponding to each peak were collected, lyophilized, and resuspended, with concentrations estimated assuming a 1:1 M conversion from the starting material. Peak “*a*” (DB33-Y-*a*) retained antimicrobial activity comparable to the parent DB33-Y mixture, whereas peaks *b*–*d* showed minimal or no activity (Fig. 6*B*), indicating that we could isolate a principle active component from the mixture of breakdown products. Mechanistic testing demonstrated that DB33-Y-*a* displaced EtBr from gDNA, with complete displacement observed at approximately 200 μM (Fig. 6*C*). Although absolute concentrations remain approximate due to the assumed molar conversion, the combined antimicrobial and intercalation data identify DB33-Y-*a* as the principal active component of DB33-Y.

Mass spectrometry of DB33-Y-*a* (Fig. S11*A*) was performed to characterize the active antimicrobial component. However, we were unable to confidently assign structures to the major peaks observed. To determine whether DB33-Y-*a* represented a single purified product, the isolated peak was reinjected onto HPLC. Additional peaks were again observed despite starting from a single collected fraction, indicating the continued formation of breakdown products rather than a stable, discrete peak/compound (Fig. S11*B*). This process was repeated a second time, and the same pattern was observed where even after two rounds of isolation, the reinjected peak consistently regenerated the same additional peaks. The solvent alone was also analyzed to confirm that these signals were not derived from the solvent itself. This instability and the presence of multiple breakdown products were consistent with our NMR observations, where the compound also appeared highly reactive and difficult to isolate as a single discrete species. This is likely due, at least in part, to the presence of dimethyl sulfoxide (DMSO), which can facilitate the photoredox activity of conjugated fluorene derivatives and promote photooxidative conversion through the generation of reactive oxidative intermediates (52, 53).

To reduce this effect, HPLC was repeated for DB33-Y (Fig. S12*A*) by resuspending the compound in acetonitrile alone and using a gradient ending in 100% water, rather than the previous method containing DMSO and ending in 95% acetonitrile. As previously observed during photoconversion experiments, conversion from the red parent compound to the yellow product proceeds much more rapidly in the presence of DMSO than in aqueous conditions. We therefore hypothesized that increasing the aqueous content and minimizing DMSO exposure would slow continued decomposition and allow isolation of cleaner products.

Under these conditions, two major peaks were observed past the solvent front, both eluting at a similar position to DB33-Y-*a* relative to the other chromatographic peaks. Mass spectrometry was performed on both fractions, and interestingly, both produced the same major species with an *m/z* of 337.1040 to 337.1044 (Fig. S12, *B* and *C*). We were able to propose a plausible structure matching this mass, consistent with a highly oxidized form of DB33-R (structure shown in Fig. S12, *B* and *C*). The presence of two distinct chromatographic peaks with the same major mass is also consistent with this assignment, as the proposed oxidized structure could exist as different stereoisomers that would be separable by chromatography while producing the same mass spectrum. Although this assignment remains tentative due to the continued reactivity of the compound, it further supports the conclusion that photoconversion generates multiple oxidative products rather than a single stable antimicrobial species.

A similar analysis was performed for DB10-Y where HPLC separation revealed three major peaks (Fig. S13*A*). The fraction that eluted at the same time as DB33-Y-*a* (DB10-Y-*a*) retained antimicrobial activity (Fig. S13*B*) and intercalative capabilities (Fig. S13*C*), whereas the remaining fractions were inactive, consistent with what was seen for DB33-Y.

Since only a principle active peak from HPLC was identified, subsequent experiments were performed using the DB33-Y mixture rather than the isolated DB33-Y-*a* fraction. The mixture produced comparable antimicrobial and DNA-intercalative activity while avoiding the additional variability and handling associated with purification of the individual compound.

### DB33-Y effectively clears intracellular bacterial reservoirs

**Figure 7:**
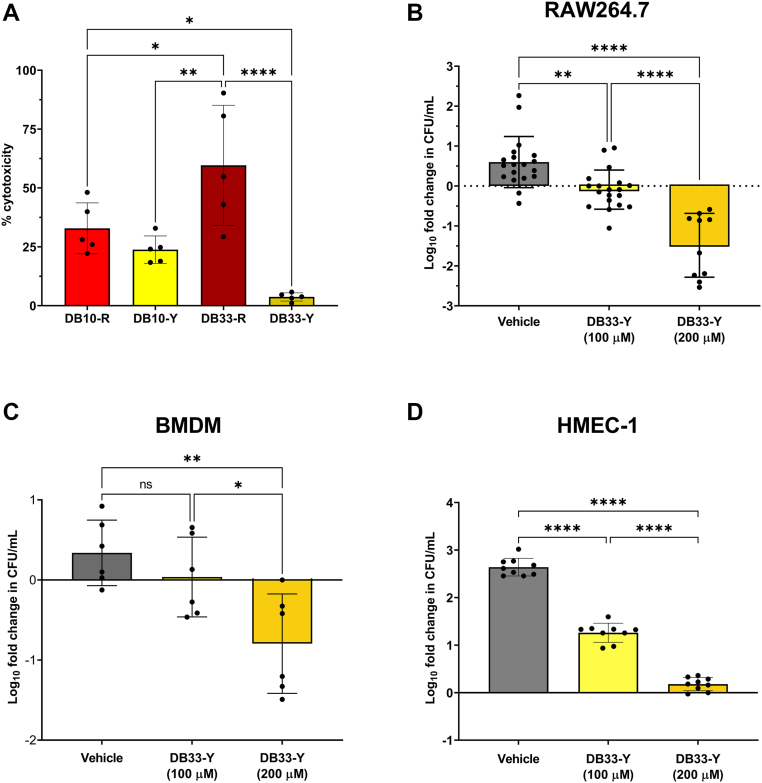
**DB33-Y effectively clears intracellular bacterial reservoirs.***A*, cytotoxicity of DB10 and DB33 at 100 μM in RAW264.7 cells, measured *via* a lactate dehydrogenase (LDH) release assay. Cells (∼70–80% confluency) were treated with DB33 or DB10 for 24 h. LDH activity was quantified using a formazan-based reaction, with absorbance measured at 500 nm. Cytotoxicity was calculated using the formula: % Cytotoxicity = [(DB10/33) – vehicle)/(tergitol – vehicle)] × 100 where tergitol is the 100% lysis control. DB33-Y was added to *S. aureus*–infected (*B*) RAW264.7 macrophages, (*C*) bone marrow-derived macrophages (BMDM) or (*D*) HMEC-1 endothelial cells at 1.5 h postinfection (hpi). Cells were lysed at 12 hpi in (*B*) and (*C*) and 8hpi in (*D*), and intracellular CFUs were quantified to assess the ability of DB33-Y to inhibit bacterial replication. All data shown represent the mean ± SD of three independent experiments. ∗*p* ≤ 0.05, ∗∗*p* ≤ 0.01, ∗∗∗∗*p* ≤ 0.0001 by one-way ANOVA with Dunnett’s multiple comparisons. CFU, colony-forming units.

*S. aureus* is a pathogen capable of persisting inside host cells (*e.g.* macrophages, dendritic cells, and endothelial cells) which can protect the bacteria from immune responses and many antibiotics (54, 55, 56). Therefore, we sought to determine whether DB10 or DB33 compounds could be used to antagonize *S. aureus* in cellular models of infection (28, 57, 58, 59, 60, 61, 62). As a first step toward this, we evaluated the cytotoxicity of these compounds using the lactate dehydrogenase (LDH) release assay, which measures LDH released from damaged mammalian cells as an indicator of membrane integrity and cellular toxicity (63). Released LDH converts lactate to pyruvate, generating NADH, which reduces a tetrazolium salt to produce the colored formazan product (63). Formazan production after DB10 or DB33 exposure was quantified spectrophotometrically at 500 nm. Notably, despite similar antibacterial activity and a shared intercalative mechanism, DB33-Y demonstrated significantly reduced cytotoxicity compared to both forms of DB10 (Fig. 7*A*). DB33-Y caused less LDH release than DB33-R at the same concentration, and substantially less than either DB10 form, highlighting its improved safety profile. To confirm that the active component, peak *a*, of both DB33-Y and DB10-Y was responsible for the observed cytotoxicity profiles and that the mixture was not masking these effects through dilution, we tested peak *a* isolated from each compound.

Given DB33-Y showed reduced cytotoxicity compared to DB10-Y, we selected it for further *in cellulo* testing. To evaluate whether DB33-Y could eradicate intracellular *S. aureus*, we infected RAW264.7 macrophages, bone marrow-derived macrophages (BMDMs), and human microvascular endothelial cells (HMEC-1) and treated cells with DB33-Y. Macrophages and epithelial cells represent key intracellular niches that may be occupied by *S. aureus* during systemic and/or localized infections (54, 55, 56). Here, gentamicin protection assays were performed as previously described (64). After gentamicin treatment to kill all extracellular non-phagocytosed bacteria, infected cells were either exposed to vehicle control or DB33-Y at either 100 or 200 μM for approximately 12 h at which time the bacterial burden was determined. At 100 μM, DB33-Y reduced intracellular *S. aureus* levels in all three cell types compared to vehicle control (Fig. 7, *B*–*D*). At 200 μM, intracellular killing was slightly more effective; however, given that this concentration was slightly cytotoxic (Fig. S14*A*), the killing of host cells is likely influencing bacterial counts. Despite this, these results indicate that DB33-Y is effective at clearing intracellular bacteria at concentrations not toxic to host cells.

### DB33-Y reduces lesion size and bacterial burden in a murine skin infection model

**Figure 8:**
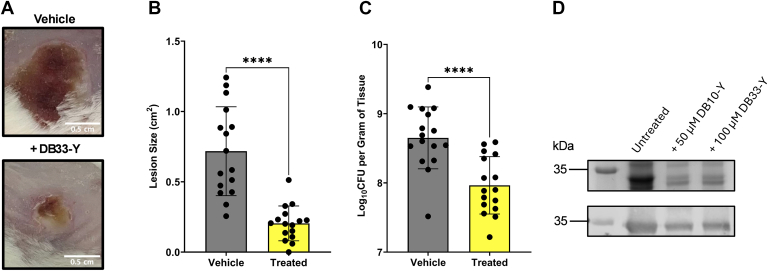
**DB33-Y treatment reduces bacterial burden and lesion size in a skin infection model.***A*, representative images of skin lesions at 3 days postinfection (dpi). Bacteria were mixed with vehicle or DB33-Y immediately prior to injection into mice. Mice were thus subcutaneously coinjected with *S. aureus* USA300 LAC (∼5 × 10^7^ CFU) and either 100 μM DB33-Y or vehicle control, as indicated, followed by a second DB33-Y dose (100 μM), or vehicle control, at one dpi. Mice were sacrificed at three dpi and lesion areas were calculated (*B*). Bacterial burdens in the excised skin tissue posthomogenization were also calculated (*C*). Data represent the mean ± SD of three independent experiments. ∗∗∗∗*p* ≤ 0.0001 by one-way ANOVA with Dunnett’s multiple comparisons. (*D*) Coomassie-stained gel (*top*) and Western blot (*bottom*) showing Hla protein levels following treatment with DB10-Y and DB33-Y at their respective MICs (50 μM and 100 μM). Protein size was determined based on comparison to BLUeye protein ladder (FroggaBio). CFU, colony-forming units.

Next, we sought to assess whether DB33-Y would display activity against *S. aureus in vivo*. To this end we employed a murine skin infection model where *S. aureus* can survive and replicate and cause dermonecrotic lesions. Using this model we evaluated the impact of DB33-Y treatment on lesion size and *S. aureus* tissue burden as compared to vehicle control treated animals. Mice were coinjected subcutaneously with USA300 LAC and DB33-Y (100 μM final concentration) or vehicle, followed by a subsequent injection at 24 h post-infection (hpi). At 72 hpi, lesion sizes were significantly smaller in the DB33-Y treated group as compared to control treated animals (Fig. 8, *A* and *B*). While bacterial burden was also significantly reduced (Fig. 8*C*), the decrease in CFU was modest relative to the reduction in lesion size. This disparity prompted us to explore whether DB33-Y might function, at least in part, through attenuation of virulence rather than by complete bacterial clearance alone.

The *hla* gene encodes α-hemolysin, a pore-forming cytotoxin that contributes to host cell lysis and tissue destruction in *S. aureus* skin infections (65). Supporting this antivirulence hypothesis, analysis of secreted proteins by both Coomassie staining and Western blot revealed reduced α-hemolysin levels in both DB10-Y and DB33-Y treated bacteria (Fig. 8*D*). This mirrors findings from our previous RNA-seq data with DB10-Y, where *hla* expression was also downregulated. Notably, the full SDS-PAGE gel (Fig. S14*B*) shows that additional secreted proteins are also differentially expressed in response to DB10-Y and DB33-Y, indicating broader effects on the bacterial secretome. Collectively, these results suggest that DB33-Y may mitigate tissue damage by suppressing key virulence determinants such as α-hemolysin, alongside other secreted factors.

## Discussion

*S. aureus* is a major human pathogen responsible for a wide range of diseases, including skin and soft tissue infections, pneumonia, and bacteremia (1, 66). The rise of MRSA has significantly complicated treatment, as these strains are resistant not only to β-lactam antibiotics but also to multiple other drug classes (67). This growing resistance has underscored the urgent need for novel antimicrobial agents, particularly those that act through mechanisms distinct from conventional antibiotics.

In looking at hits from a previously conducted high-throughput chemical screen (27, 28), we noted that approximately 11% of compounds active against MRSA contained planar, aromatic moieties—structural features commonly associated with DNA intercalation (9). Although such structures are often deprioritized due to concerns over mammalian genotoxicity (21, 22), several, including doxorubicin, have demonstrated potent antimicrobial activity (8, 67). Among the few reproducibly active hits, we prioritized DB10, which contains a fluorene core, for its potent antibacterial activity and unique photochemical properties.

Notably, we observed that upon prolonged ambient light exposure DB10 underwent a spontaneous color change from red (DB10-R) to yellow (DB10-Y), a process we confirmed to be driven specifically by UVA (365 nm) irradiation. UV-Vis spectra revealed a sharp isosbestic point indicating a clean, two-species conversion between DB10-R and DB10-Y, rather than nonspecific photodegradation. Moreover, NMR analysis confirmed that this is a one-way structural rearrangement rather than a reversible isomerization. This irreversible transformation distinguishes DB10 from classical photoswitchable antibiotics, which typically rely on reversible cis–trans isomerization of functional groups such as azobenzenes or stilbenes (68, 69). Furthermore, unlike conventional photoswitches which often necessitate fluorophore conjugation with traditional antibiotics or rely on photodynamic ROS generation (70, 71), DB10 exhibited antibacterial activity independently of these features. Importantly, both DB10-R and DB10-Y possess unique chemical structures that, to our knowledge, have not been previously characterized in the literature.

Photoconversion of DB10-R to DB10-Y involves the loss of the hydrophobic *N*-methylaniline group and formation of a polar aldehyde, resulting in enhanced aqueous solubility and slightly reduced cytotoxicity against RAW264.7 macrophages. Notably, this structural shift is also accompanied by a slight reduction in the MIC across most Gram-positive species tested. Although both forms are ineffective against Gram-negative bacteria, likely due to outer membrane impermeability which is a common barrier to DNA intercalating agents (6), they show broad activity against Gram-positive pathogens. However, certain species, including *S. cohnii*, *S. warneri*, and *B. subtilis*, remained less susceptible. This reduced efficacy may reflect species-specific differences in membrane permeability or efflux, but it is also possible that DB10 compounds exhibit sequence-specific DNA binding, and that the relevant target motifs are less prevalent or accessible in these bacterial genomes, as has been observed with other intercalators (8).

To further elucidate the functional differences following DB10 photoconversion, we performed RNA-seq analysis on USA300 LAC treated with either DB10-R or DB10-Y. Both treatments reduced expression of key virulence factors, including *lukD* (a leukotoxin that lyses immune cells) (72), *hla* (an alpha-hemolysin that damages host membranes) (73), and the *spl* operon (serine proteases involved in immune evasion and tissue dissemination) (74). Western blots confirmed reduced Hla protein levels after DB10-Y or DB33-Y treatment. Repression of these virulence genes after antibiotic exposure has been well-established and may reflect a general stress response or disruption of global regulators (28, 72, 73, 75).

DB10-R induced upregulation of *clpB*, *clpC*, *mcsA*, and *mcsB* which are components of the Clp proteostasis network that aid in refolding and degradation of misfolded proteins during stress (32, 76). Also upregulated was *umuC*, an error-prone polymerase typically activated during DNA damage (77). Notably, canonical SOS regulators *recA* and *lexA* (7) were not induced, in contrast to antibiotics like mitomycin C and ciprofloxacin, which activate the SOS cascade *via* RecA-dependent LexA cleavage (77, 78).

DB10-Y elicited a similar, but distinct, RecA-independent stress response. While *clpB* and *clpC* were again upregulated, though slightly below statistical cut-off, DB10-Y-treated cells showed increased expression of *rusA* and *ssb*, which are genes involved in recombinational DNA repair. *rusA* encodes a Holliday junction resolvase capable of resolving recombination intermediates (35, 77), while *ssb* stabilizes single-stranded DNA at stalled replication forks (36). These genes are not part of the RecA-dependent SOS response, implying activation of alternative repair pathways, potentially resembling single-strand annealing, a RecA-independent mechanism for resolving double-strand breaks (DSBs) (33, 79).

We next tested whether DB10-Y interacts directly with DNA. DB10-Y displaced EtBr, indicating intercalation, and caused DSBs in bacterial cells. In contrast, no damage was observed when purified gDNA was exposed under the same conditions, suggesting that the breaks arise indirectly through cellular processes, such as replication or transcription interference, as has been seen for other planar aromatic compounds, rather than through direct chemical cleavage (80, 81). Furthermore, while DB10-Y was found to displace EtBr and intercalate into mammalian DNA, intercalation alone does not necessarily induce damage. Many small molecules bind DNA without stabilizing cleavage complexes or disrupting essential enzymes, altering DNA conformation without producing lethal lesions (82, 83). Simple intercalation can therefore occur without triggering DNA breaks or cell death, consistent with our observation that DB10-Y binds mammalian DNA *in vitro*, while being minimally cytotoxic *ex vivo*.

Given the atypical activation of the DNA damage response observed with DB10-Y, we hypothesized that its mechanism of action is pleiotropic and not limited to classical SOS-mediated pathways. To further investigate this, we attempted to evolve resistance through serial passaging. However, only low-level resistance emerged, and only after a prolonged period of selection, suggesting that high-level resistance may be difficult to achieve, likely due to the essential nature of the DNA target.

Interestingly, whole-genome sequencing of resistant populations revealed mutations in *clpX* or the *clpX* upstream promoter region in two independent subpopulations. Although *clpX* is not likely the primary target of DB10-Y, its repeated mutation suggests a compensatory role in mitigating the cellular effects of the compound, possibly through modulation of proteostasis or stress responses. The absence of mutations in typical resistance pathways such as efflux pumps, DNA repair genes, or alterations in membrane permeability, commonly observed with antibiotics that cause genotoxic stress and DNA damage (84, 85), supports the idea that DB10-Y activates a nontraditional, RecA-independent DNA damage response, possibly engaging alternative or poorly characterized stress responses in *S. aureus*.

ClpX inactivation has previously been associated with increased resistance to β-lactams and DNA-damaging agents (46, 86). Specifically, ClpX has been shown to interact with LexA to promote its turnover and facilitate proper regulation of the SOS response, thereby influencing DNA damage repair, stress adaptation, and susceptibility to antibiotics that induce genotoxic stress (87, 88). However, the divergent phenotypes of YR-1 and YR-2, one showing increased ampicillin resistance and the other increased susceptibility to vancomycin and ofloxacin, argue against a full loss of ClpX function. The lack of broad cross-resistance, combined with unaltered DB10-Y sensitivity in *clpP* mutants suggests resistance arose from altered regulation of *clpX*, rather than complete inactivation. Supporting this, overexpression of the mutant *clpX* alleles reversed the altered susceptibility phenotypes observed, likely by disrupting proteostasis or interfering with stress adaptation networks. Taken together, the RNA-seq data, DNA intercalation and damage assays, and the emergence of a *clpX* SNP associated with resistance all support DNA intercalation as the primary mode of action of DB10/33, with downstream effects consistent with DNA damage stress responses.

To investigate structure–activity relationships, several DB10 analogs were tested for DNA binding and antibacterial activity. DB10-Y, DB33-Y, DB42, and DB35 all contain a diene side chain conjugated to the fluorene core, which likely enhances planarity and facilitates DNA intercalation *via* π–π stacking (89, 90). Indeed, all analogs except DB28 were capable of some DNA intercalation, though DB91 and DB55 were weak. However, only DB33 exhibited antibacterial activity, indicating that intercalation alone is not sufficient for function. Indeed, DB42 and DB35 contain nitro groups that increase polarity and may hinder membrane diffusion (91), limiting intracellular access despite good DNA affinity. In contrast, DB10 and DB33 lack strongly polar groups, likely facilitating passive uptake (92). DB55 and DB91 lacked conjugated side chains and instead contained bulky groups (*e.g.*, Br in DB55), which may disrupt planarity and reduce DNA binding. Similarly, DB38 lacks conjugation and electron-donating or -withdrawing groups, precluding effective coordination with DNA.

A closer comparison of DB33-R and DB10-R, which differ only by a terminal phenyl group, shows DB33-R has a slightly lower MIC, suggesting possible steric hindrance of DNA interaction. Based on the proposed photoconversion mechanism and HPLC separation observed, DB33-Y and DB10-Y likely both contain the same photoproducts, predicted to be the active species, yet DB33-Y had a slightly higher MIC. Although the bioactive peak eluted at the same retention time for both compounds, differences in MIC, cytotoxicity, and intercalative activity may reflect differences in the composition or relative abundance of photoproduct species within this peak.

Structural characterization of the active DB33-Y-*a* fraction was complicated by its instability. Mass spectrometry did not allow confident assignment of the major species observed, and repeated reinjection of the isolated HPLC peak consistently generated additional peaks, even after multiple rounds of purification. Similar instability was observed by NMR, indicating that the active fraction exists as a dynamic mixture of interconverting breakdown products rather than a single discrete molecule. One major ion at *m/z* 235.0002 was most consistent with an acetonitrile-associated adduct of a parent ion at *m/z* 193.9736, although the identity of this species could not be definitively assigned.

This behavior is consistent with the highly conjugated fluorene–triene scaffold of these compounds. Indeed, fluorene derivatives themselves are known to participate in photoredox processes, and fluorene-based systems have been shown to activate molecular oxygen and promote formation of ROS such as superoxide radicals (93, 94). This likely drives continued oxidation of the conjugated triene-enamine system and formation of multiple oxidative intermediates during purification. Under more aqueous HPLC conditions, the chromatographic profile simplified and one plausible highly oxidized intermediate could be assigned, whereas DMSO-containing conditions resulted in substantially greater decomposition. Together, these findings suggest that antimicrobial activity arises from a mixture of reactive photooxidation products, and that differences in MIC and cytotoxicity likely reflect variation in the relative abundance of these transient species. Future work will focus on further refining of these compounds to improve the stability.

Despite the reduced potency, DB33-Y exhibited relatively low cytotoxicity compared to DB10-Y against RAW264.7 macrophages, making it a more suitable candidate for *in vivo* evaluation. We therefore selected DB33-Y for further testing in a murine skin infection model. Interestingly, after treatment with DB33-Y, we observed only a modest decrease in bacterial CFU, yet a significant reduction in lesion size. Although DB33-Y was administered at its *in vitro* MIC (100 μM) based on testing in MHB, this concentration may not reflect the effective bactericidal dose within skin tissue, which is influenced by host absorption, immune responses, and compound distribution (95). This suggests DB33-Y may function primarily as an antivirulence agent at sub-MIC levels, which is further supported by our RNA-seq data that showed downregulation of virulence genes and reduced α-hemolysin levels following DB10-Y. Similar concentration-dependent antivirulence effects have been reported for clindamycin and tigecycline (96, 97). This antivirulence activity observed for DB10-Y and DB33-Y may result in clinical advantages by reducing tissue damage and immune activation while lowering the selective pressure for resistance (98).

Overall, DB10 and DB33 represent promising scaffolds for further antibiotic development. Modest structural changes were sufficient to modulate cytotoxicity and preserve DNA intercalation, making DB33 a more attractive molecule for further study; coincidentally, near the completion of our studies, we could no longer identify a commercial supplier of DB10. The dual bactericidal and antivirulence properties of these molecules may enable targeted therapeutic strategies depending on infection context. Collectively, the combination of *in vitro* potency against MRSA, *in vivo* efficacy in a skin infection model, and limited resistance development supports further exploration of this scaffold as a potent treatment option for MRSA.

## Experimental procedures

### Bacterial strains and plasmids

Bacterial strains and plasmids are listed in Table S2. All bacteria were cultured at 37 °C with shaking at 200 rpm, with the exception of Streptococcal species, which were grown without shaking. Most Gram-positive bacteria were grown in either TSB or MHB, unless otherwise stated. Streptococcal species were grown in Todd Hewitt broth supplemented with 1% yeast extract (THY). Gram-negative species were grown in LB broth.

### High-throughput screen

The initial high-throughput screen used to identify bioactives of interest was conducted previously (27, 28), and these results were revisited to search for alternative moieties of interest. Of the 965 initial hits, 107 contained a planar aromatic moiety, as identified using a manual search. Planar aromatic moieties identified contained benzothiophene-, indole-, naphthalene, fluorene-, chromene-, phenthrene-, and anthrone-based core moieties, all of which have been shown to be involved in DNA damage or intercalation (99, 100, 101, 102, 103). However, in a secondary screen in chemically defined medium containing glucose, only three of the initial planar-structure hits retained activity These inhibitors contained a fluorene, indole, or phenanthrene core moiety. The indole-containing inhibitor has been previously established to be a ligand that binds to estrogen receptor β and thus was screened out (104). Given the prevalence of fluorene-containing inhibitors in the initial screen (∼10%) compared to the prevalence of phenanthrene-containing inhibitors (4%), along with the lower MIC associated with the fluorene compound in the secondary screen, we chose to proceed with this compound, which we named “DB10”.

### Determination of MIC

Serial dilutions of DB10 and DB33 were prepared in MHB for Gram-positive isolates—except for *Streptococcus* species, which were grown in Todd-Hewitt broth supplemented with yeast extract (THY)—and in LB broth for Gram-negative isolates. Bacteria were then added to achieve a starting OD_600_ equivalent of 0.01. Cultures were grown overnight at 37 °C with or without shaking as required. The concentration that completely inhibited growth, as measured spectrophotometrically, was determined to be the MIC.

### Bactericidal effect of DB10

An overnight culture of USA300 LAC was washed and inoculated, at a starting OD_600_ equivalent of 0.1, into PBS containing various concentrations of inhibitor. Cultures were incubated overnight at 37 °C without shaking before being serially diluted and drop plated onto MHA. After overnight incubation of the plates at 37 °C, CFU/ml were enumerated.

### UV-based photoconversion of DB10 and DB33

DB10 or DB33 were stored as a powder away from light at −20 °C until use. Just before UV exposure, the compounds were reconstituted in 100% DMSO and subsequently diluted 3:100 in DMSO in a 96-well plate. A spectral scan ranging from 290 nm to 610 nm was conducted before UV exposure, immediately after dilution and at every 5 min after until complete conversion was observed (∼20 min). UV exposure was conducted by placing the 96-well plate on top of a handheld UVGL-25 compact UV lamp with either UVA (356 nm) or UVB (254 nm) wavelengths switched on. The absorbance readings were corrected for background using DMSO.

### Chemical characterization of the red and yellow forms of DB10 and DB33

NMR spectra for characterization were obtained in DMSO-d6 using a 600 MHz Bruker Ascend 600 instrument. NMR chemical shifts are reported in ppm and are calibrated against the residual solvent signals of DMSO-d6 (2.5 ppm). Coupling constants (J) are given in Hz. Electrospray ionization mass spectrometry was performed using anOrbitrap Exploris 120 mass spectrometer.

HPLC was performed using an Agilent 1200 HPLC System equipped with a ZORBAX Eclipse XDB-C18 column (4.6 × 150 mm, 5 μm; Agilent). Detection was carried out by UV absorbance at 360 nm. The samples were injected at 0.1 mg/ml using 25:50:50 DMSO (DB10/33-Y): acetonitrile: water and run at a gradient from 0 to 5 min, 0 to 40% ACN; 5 to 20 min, 40 to 70% ACN; 20 to 22 min, 95% ACN; and 22 to 26 min, 40% ACN.

A second attempt at HPLC was performed on DB33-Y using a Waters alliance 2695 separation module connected to a Jupiter 5 μm C18 300 Å LC Column, with a Waters 2487 dual λ absorbance detector detecting at 330 and 350 nm. The samples were injected at 4.0 mg/ml using 25:75 acetonitrile: water to 100% water as eluent over 30 min. Water was used in this run over DMSO in an attempt to stabilize the photoconversion and oxidative products.

#### Effect of irradiation on 10 g/L DMSO solution of DB10 examined by NMR spectroscopy

DB10 (4.5 mg) was dissolved in 0.5 ml of DMSO-d6. The sample was irradiated using a 460 nm blue light for 44 h (36 W LED) at 21 °C and ^1^H NMR spectra were recorded at various time intervals.

#### Effect of irradiation on 9 g/L DMSO solution of DB33 examined by NMR spectroscopy

DB33 (4.5 mg) was dissolved in 0.5 ml of DMSO-d6. The irradiation with UV light (365 nm) was performed using a 10 W LED light source with 1 A constant current, 400 Hz pulse for 15 h. Next, the sample was irradiated using a 450 nm blue light for 7.8 h (36 W LED), followed by 8.2 h of further irradiation while bubbling air into the sample *via* a needle. The solution was incubated at 21 °C and 1H NMR spectra were recorded at various time intervals over the cumulative 31 h of irradiation. The sample was then separated using HPLC. The bioactive fraction (DB33-Y-*a*) was collected and analyzed by mass spectroscopy.

Characterization of compound 1 (DB33): 1H NMR (600 MHz, DMSO) δ 8.04 (d, J = 7.5 Hz, 1H), 7.88 (d, J = 7.3 Hz, 1H), 7.82 (d, J = 7.3 Hz, 1H), 7.78 (d, J = 7.5 Hz, 1H), 7.41 (d, J = 12.3 Hz, 1H), 7.37 – 7.20 (m, 4H), 6.97 (t, J = 12.0 Hz, 1H), 6.92 – 6.82 (m, 2H), 5.45 (t, J = 12.0 Hz, 1H), 2.89 (s, 6H). 13C NMR (151 MHz, DMSO) δ 149.6, 146.0, 140.3, 138.8, 137.6, 136.8, 132.2, 126.9, 126.7, 126.0, 125.7, 125.0, 124.2, 120.2, 120.0, 119.5, 116.7, 100.0, 40.5. HRMS: calcd [M]+ (C20H19N): 273.1517 Found (ESI): 273.1510.

### RNA sequencing

USA300 LAC was subcultured into TSB to a final OD_600_ of 0.1 and grown until early exponential phase (OD600 ∼ 1.5). Either vehicle control or sub-MIC DB10-R (50 μM in TSB) or DB10-Y (50 μM in TSB) was then added and the cultures were grown for an additional hour. Cells at an OD_600_ equivalent of three were combined with an equal volume of RNA-protect Bacterial Reagent (Qiagen) and pelleted before being frozen at −80 °C overnight. Frozen pellets were thawed and resuspended in 750 μl of TE buffer (pH 8.0) and 50 μl of 1 mg/ml lysostaphin and then incubated at 37 °C for 1 h. RNA was extracted using 2 ml of TRI Reagent (Sigma-Aldrich) and 500 μl of BCP (Sigma-Aldrich). After mixing, the aqueous layer was added to an equal volume of ice-cold isopropanol, and the samples were then incubated overnight at −20 °C. Samples were pelleted at 4 °C for 20 min at 11,000*g*, resuspended in 500 μl of ice-cold 75% ethanol and pelleted again. Pellets were dried and resuspended in 86 μl of nuclease-free ddH_2_O before treatment with Invitrogen Turbo DNase for 1 h, according to the manufacturer’s instructions. RNA was precipitated using a phenol-chloroform extraction. RNA-sequencing and initial data analysis using transcripts per million comparisons were conducted by SeqCenter. Further analyses were conducted using the Geneious Prime software package.

### DNA intercalation and damage

DNA used for intercalation and damage assays was isolated from USA300 LAC using phenol-chloroform extractions. DNA from RAW264.7 macrophages was isolated using the PureLink gDNA Mini Kit from Invitrogen according to the manufacturer’s instructions.

To determine the DNA intercalative ability of DB10-Y and DB33-Y, an EtBr displacement assay was performed as described previously (15), with some modifications. Briefly, EtBr was added to PBS in a 96-well plate to a final concentration of 0.6 μM and gDNA was added at a final concentration of 20 ng/μl. Samples were briefly incubated at room temperature for 5 min to allow the EtBr to intercalate into DNA before various concentrations of DB10-Y and DB33-Y were added. The plate was then incubated at room temperature in the dark for 30 min. Fluorescence spectra (530–700 nm) of samples was measured using a Synergy H4 with an excitation of 525 nm, corresponding to the excitation of EtBr. Wells were background corrected using PBS or DB10-Y/DB33-Y with DNA in the absence of EtBr, as appropriate.

A neutral and alkaline/neutral assay was used to visualize double- and single-strand DNA breaks (DSBs and SSBs respectively) in USA300 LAC gDNA using previously described methods (42), with modifications. Briefly, USA300 LAC was subcultured into fresh TSB containing various concentrations of DB10 or ERY. The cultures were incubated overnight at 37 °C with shaking before DNA was phenol-chloroform extracted. To visualize DSBs, gDNA was diluted to a concentration of 500 ng/μl in TE buffer. To visualize alkaline unwinding-sensitive sites corresponding to SSBs, 3 μl of 1 M Na_2_HPO_4_ were added to 20 μl of TE buffer containing 500 ng of gDNA to induce unwinding. 9 μl of 0.1 M HCl was then immediately added to the sample, mixed over ice, and allowed to sit for 4 min to allow for DNA renaturation. Loading buffer (0.25% bromophenol blue, 60% glycerol) was added to both conditions, and the samples were run on a 0.8% Tris/Borate/EDTA (TBE) gel with SybrSafe for DNA visualization.

### Detection of superoxide formation

Superoxide formation by USA300 LAC cells after DB10-Y exposure was measured as previously described (28), with modifications. Briefly, DB10-Y or a DMSO vehicle control was added to 100 μl of USA300 at an OD600 of 0.1 in Hank’s Balanced Salt Solution. 500 μl of NBT (1 mg/ml) was added to the samples and incubated at 37 °C for 60 min. The reaction was stopped by adding 100 μl of 0.1 M HCl and the samples were spun at 5000 rpm for 15 min. Pellets were resuspended in equal volumes of DMSO and Hank’s Balanced Salt Solution. The absorbance levels of the solutions were then read at 575 nm to quantify intracellular ROS production.

### Evolution and characterization of resistant mutants

#### Evolutionary adaptation

Overnight cultures of USA300 LAC were subcultured 1:100 daily in TSB with 0.5x MIC DB10-Y (50 μM). MICs were taken periodically to evaluate resistance evolution. After 39 days low-level (2-fold increase) resistance was observed. The culture was passaged for an additional 7 days to ensure stability. The resultant culture was plated on agar to isolate individual colonies, and colonies were patched onto either 5% sheep’s blood agar, or standard method caseinate agar (105) to determine hemolytic and proteolytic capabilities. Two colonies with distinct phenotypes based on the blood and caseinate agar were selected and designated YR-1 and YR-2.

#### Sequencing of resistant mutants

gDNA was isolated from YR-1 and YR-2 using phenol chloroform extraction and sequenced at SeqCenter. DNA sequence reads were paired and mapped to the reference USA300_FPR3757 genome (CP000255.1) using the Geneious software package. Variant analysis was completed using FreeBayes.

#### Cloning and overexpression of mutant ClpX

gDNA was isolated from USA300 LAC by phenol-chloroform extraction. Primers containing a *Kpn*I or *Sac*I restriction site were used to amplify *clpX* from either YR-1 or YR-2 (Table S3 for primers). The amplified product was ligated into pALC2073 that was also digested using *Kpn*I and *Sac*I. The ligated product was transformed into *E. coli* DH5α. Sequence-confirmed recombinant plasmids were isolated from *E. coli* and electroporated into RN4220. Phage lysate was prepared from the resultant RN4220 strain carrying the p*clpX-*YR-1 and YR-2 plasmids using phage 80α and lysates were used to transduce recipient strains YR-1 and YR-2 with recombinant plasmids with selection on chloramphenicol-containing solid media.

### TCA precipitation of secreted proteins

Cultures of USA300 LAC were subcultured into fresh TSB containing DB10-Y or DB33-Y at various concentrations and cultures were grown to stationary phase overnight. Secreted proteins were precipitated using trichloroacetic acid. Briefly, supernatant from an OD_600_ equivalent of six of culture was added to an equal volume of trichloroacetic acid and incubated at 4 °C overnight. Proteins were washed twice with ice-cold 70% ethanol and dried at 37 °C before being resuspended in 1 × Laemmli SDS-PAGE reducing buffer and boiled for 5 min at 100 °C. Proteins were loaded onto a 12% SDS-PAGE gel and run at 115V for 10 min, then 150V for 55 min. Gels were stained with Coomassie for 1 h and then destained overnight before visualization.

### Western blot

Western blot analysis of Hla was conducted by transferring the SDS-PAGE-resolved proteins to a nitrocellulose membrane. The membrane was blocked in PBS containing 0.1% (v/v) Tween 20 and 5% (w/v) skim milk powder. Following blocking, the membrane was incubated overnight with a rabbit polyclonal anti-Hla antibody (1:1000; Sigma-Aldrich), then washed three times with PBS-Tween 20. A donkey anti-rabbit IRDye 800 secondary antibody (1:20,000; Rockland) was applied for 1 h, followed by three PBS-Tween 20 washes. Imaging was performed using an Odyssey CLx system (Li-Cor Biosciences).

### Lactate dehydrogenase (LDH) release

Quantification of cytotoxicity using LDH release was done using the LDH Cytotoxicity Assay Kit (Cat #MAK529) from Sigma-Aldrich. The manufacturer’s instructions were followed, with modifications. Briefly, RAW264.7 macrophages were seeded into a 96-well plate and grown to a confluency of approximately 60% in RPMI + 5% fetal bovine serum (FBS). Media was removed and replaced with fresh media containing various concentrations of either DB10, DB33, a DMSO vehicle control or a tergitol positive control. Cells were incubated at 37 °C for 24 h before 150 μl of Reagent was added to each well and the plate was incubated at room temperature for 10 min before formazan production corresponding to LDH release was read at 500 nm. Cytotoxicity was calculated using the following equation: Cytotoxicity= (OD_Sample_-OD_Control_)/(OD_Total Lysis_-OD_Control_) × 100 (%). The plate was read before addition of Reagent and these values were used for background correction.

### Treatment of macrophage and endothelial cell infections

The efficacy of DB33-Y against intracellular *S. aureus* reservoirs was assessed using procedures as previously described (64). RAW264.7 macrophages were grown in RPMI + 5% FBS, HMEC-1 cells were grown in MCDB 131 supplemented with 10 mM L-Glutamine, 10 ng/ml epidermal growth factor (EGF), 1 μg/ml hydrocortizone and 10% (v/v) FBS and bone marrow-derived macrophages (BMDMs) were grown in RPMI + 10% FBS. For all cell lines cells were seeded into 12-well plates 24 h prior to infection. Infections were performed in serum-free media until gentamicin treatment, at which serum-containing media was reintroduced. USA300 LAC was used to infect RAW 264.7 macrophages, BMDMs, and HMEC-1 cells at a multiplicity of infection of 10. After a 0.5-h infection period, 100 μg/ml gentamicin was added to wells and incubated for 1 h to kill all extracellular bacteria, before cells were gently washed to remove gentamicin. DB33-Y was then added to the culture media to evaluate its ability to restrict intracellular bacterial growth. At 12 h post-infection (hpi) for RAWs and BMDMs and eight hpi for HMEC-1s, cells were lysed in 0.1% (v/v) Triton X-100 in sterile PBS. Lysates were plated on TSA for CFU enumeration and compared to a control sample lysed at 1.5 hpi (immediately after gentamicin treatment) to assess intracellular bacterial replication. Fold change was calculated by dividing CFUs at 12 or 8 hpi by CFUs at 1.5 hpi.

### Mice infections

The mouse study (Animal Use Protocol #2021-090) was approved by the University of Western Ontario Animal Care Committee. Eight-to-ten-week-old male and female BALB/c mice were shaved and depilated using Nair 1 day prior to infection. Overnight cultures (grown in TSB) of *S. aureus* USA300 were subcultured at an OD600 of 0.1 in TSB and grown to an OD600 of 2.0 to 2.2. Bacterial cells were pelleted and washed in PBS twice. Bacterial cells were then normalized to an OD600 equivalent of 3.7 and 25 μl of the bacterial suspension were mixed with either 25 μl of 200 μM B33 (equivalent to a working concentration of approximately ∼ 1 x 10ˆ9 CFU/ml, and 100 μM, respectively) or 25 μl of PBS with vehicle. This mixture was immediately injected subcutaneously into the rear flanks of each animal. This dosage was chosen based on the *in vitro* MIC of B33 in MHB. At 24 hpi, either 50 μl of 100 μM B33 or 50 μl of PBS with vehicle were injected into the infection sites. Infected mice were monitored daily for 3 days and sacrificed at 72 hpi. Lesions were excised in PBS with 0.1% (v/v) Triton X-100, homogenized in a bullet blender tissue homogenizer (2 × 5 min at speed 6) and serially diluted before being plated onto MSA to enumerate CFUs. Lesion sizes were analyzed in ImageJ.

## Data availability

RNAseq data can be found in the GEO repository under accession number GSE303558. The authors declare that the data supporting the findings of this study are available within the paper and its supporting Supplementary Information files.

## Supporting information

This article contains supporting information; Figures S1–S13 and Tables S1–S5 (106, https://webbook.nist.gov/cgi/cbook.cgi?ID=C7235407).

## Conflict of interest

The authors declare that they have no conflicts of interest with the contents of this article.
