# Supplementary material for: Photochemical modification of two fluorene-based molecules with DNA intercalating and anti-methicillin resistant Staphylococcus aureus activity
Source: J Biol Chem. 2026 May 8;302(6):113133. doi: 10.1016/j.jbc.2026.113133 (PMC13255072; doi:10.1016/j.jbc.2026.113133)
Supplement: Supporting Figures and Tables [file mmc1.docx]

**Supplementary Information**

**Figure S1. ^1^H NMR spectrum of DB10 in DMSO-*d6* while exposed to 460nm light.** The disappearance of the peak at 6 ppm (**b**) by 44 h is evidence of complete photoconversion while the peak at ~8.5 ppm is evidence of aldehyde formation.


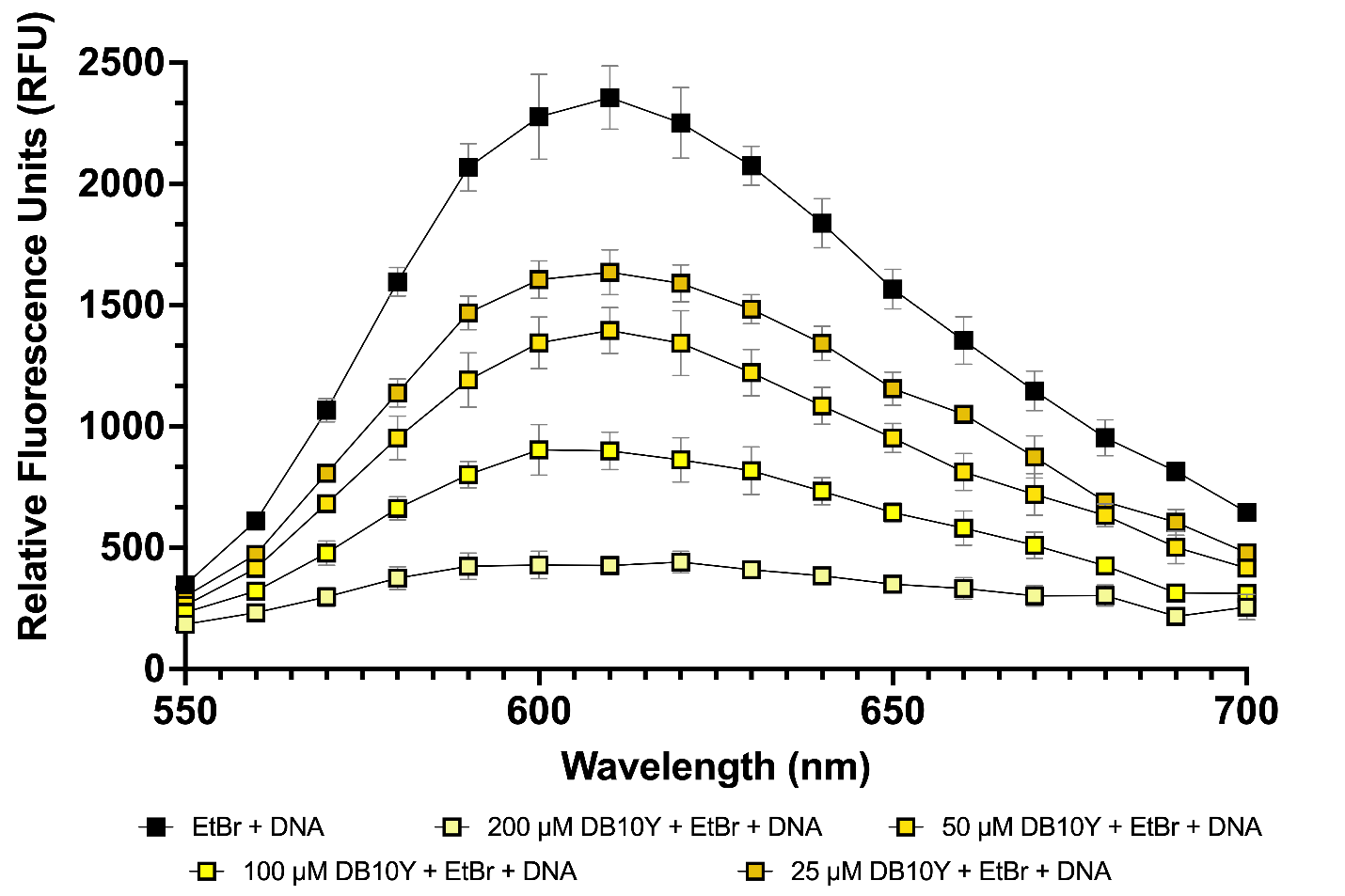


**Figure S2. DB10-Y intercalates into mammalian DNA *in vitro*.** EtBr, DNA (isolated from RAW264.7 cells) and various concentrations of DB10-Y or the control erythromycin were incubated together for 30 minutes in the dark before the fluorescence spectra of EtBr were read (excitation 525 nm). DB10-Y, EtBr and DNA alone showed no intrinsic fluorescence, and ERY, a non-intercalating control, had no effect on EtBr fluorescence. Data are shown as the mean ± SD from at least three biological replicates.

**Figure S3. Exposure of *S. aureus* to DB10-Y results in non-RecA-mediated DNA damage response.** (**A**) Neutral/alkaline treatment of genomic DNA isolated from *S. aureu*s cultures grown in the presence of either DMSO vehicle control, or DB10-Y at the indicated concentrations. Shown is a representative gel of three biological replicates, and each lane within a sample group is a technical duplicate. Samples were run through a 0.8% Tris/Borate/EDTA (TBE) gel and the ladder for all gels was a 1kB DNA ladder (FroggaBio). (**B**) Intracellular superoxide (O2^•−^) levels in USA300 LAC after exposure to DB10-Y for 1h. Nitro-blue tetrazolium (NBT) reduction was used to determine O2^•−^ levels. (**C**) MIC of USA300 LAC compared to mutants with transposon insertions in genes, as indicated, involved in the canonical DNA damage response. Data are shown as the mean ± SD of at least three independent experiments.


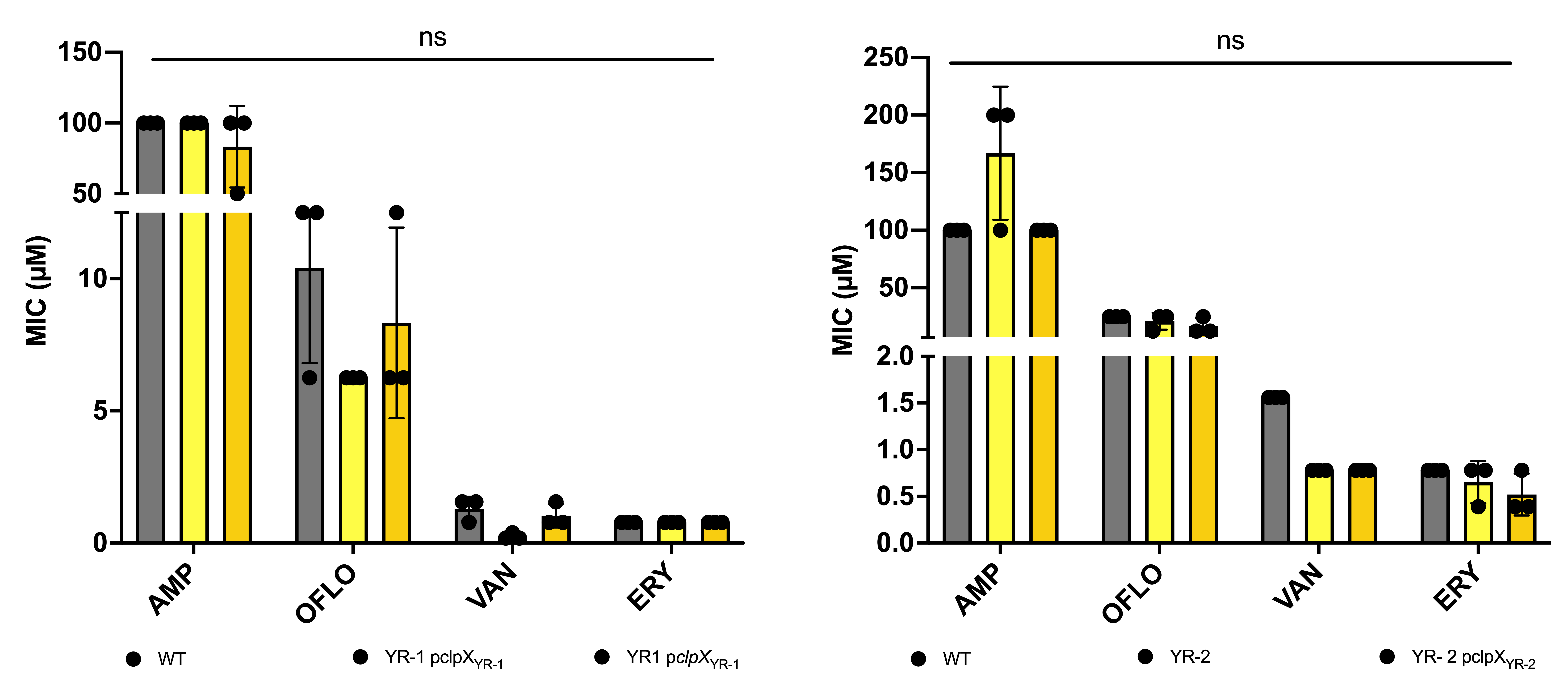


**Figure S4. Overexpression of *clpX* alters bacterial susceptibility to classical antibiotics.** MICs of (**A**) YR-1 and (**B**) YR-2 compared to WT USA300 LAC and their corresponding *clpX* overexpression strains against antibiotics previously associated with *clpX*-linked resistance (AMP) and antibiotics involved in DNA damage (ofloxacin (OFLO) and vancomycin (VAN)) and downstream effects (ERY).

**Figure S5. Structures of analogs of DB10.** Compounds with biological activity are labelled in red, and compounds without biological activity are labelled in black.


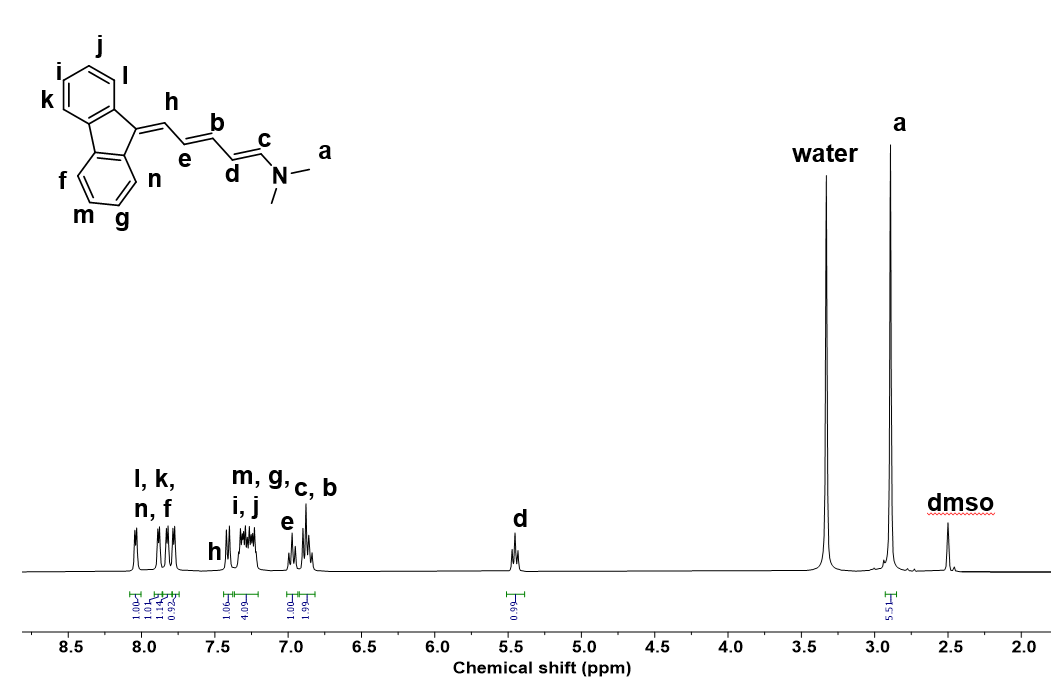


**Figure S6. ^1^H NMR spectrum of DB33 (600 MHz, DMSO-*d_6_*)**


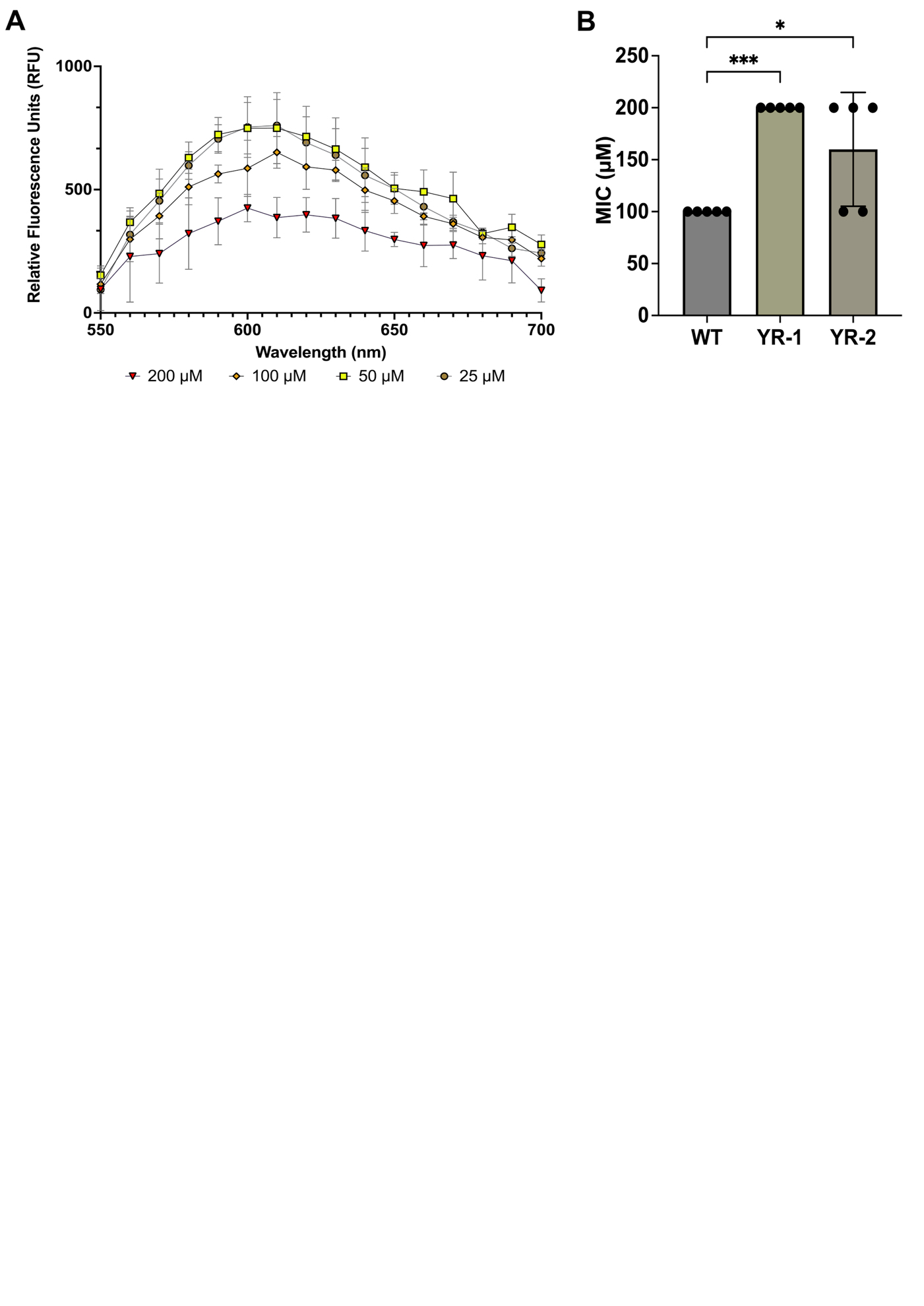


**Figure S7. Intercalation of DB33-Y into DNA *in vitro*.** (A) Like DB10-Y, DB33-Y intercalates into DNA, as shown by its ability to displace ethidium bromide (EtBr) from DNA, as measured by the drop in fluorescence. EtBr, DNA, and various concentrations of DB33-Y were incubated for 30 minutes in the dark before EtBr fluorescence spectra were measured (excitation at 525 nm). DB33-Y, EtBr, and DNA alone exhibited no intrinsic fluorescence. Data are presented as mean ± SD from three biological replicates. (B) DB33-Y exhibits reduced activity against *clpX* mutant strains YR-1 and YR-2, which were originally selected through enhanced resistance to DB10-Y.


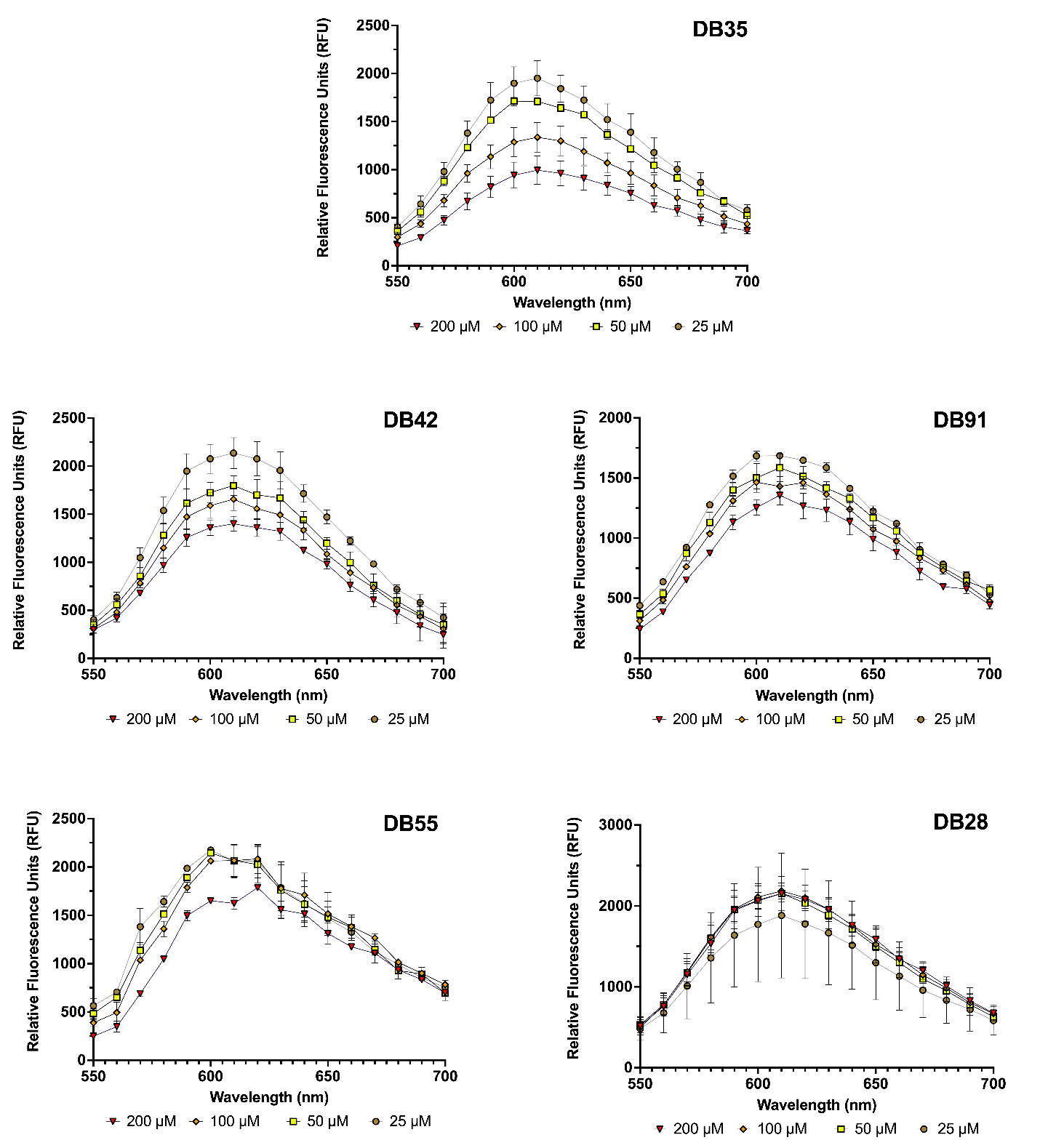


**Figure S8. Most DB10 analogs tested are DNA intercalators.** EtBr, DNA and various concentrations of analogs were incubated together for 30 minutes in the dark before the fluorescence spectra of EtBr was read (excitation 525 nm). DB35 and DB42 displaced EtBr at approximately the same rate as DB33-Y, DB91 and DB55 only slightly displaced EtBr at 200 μM. DB28 did not displace EtBr.


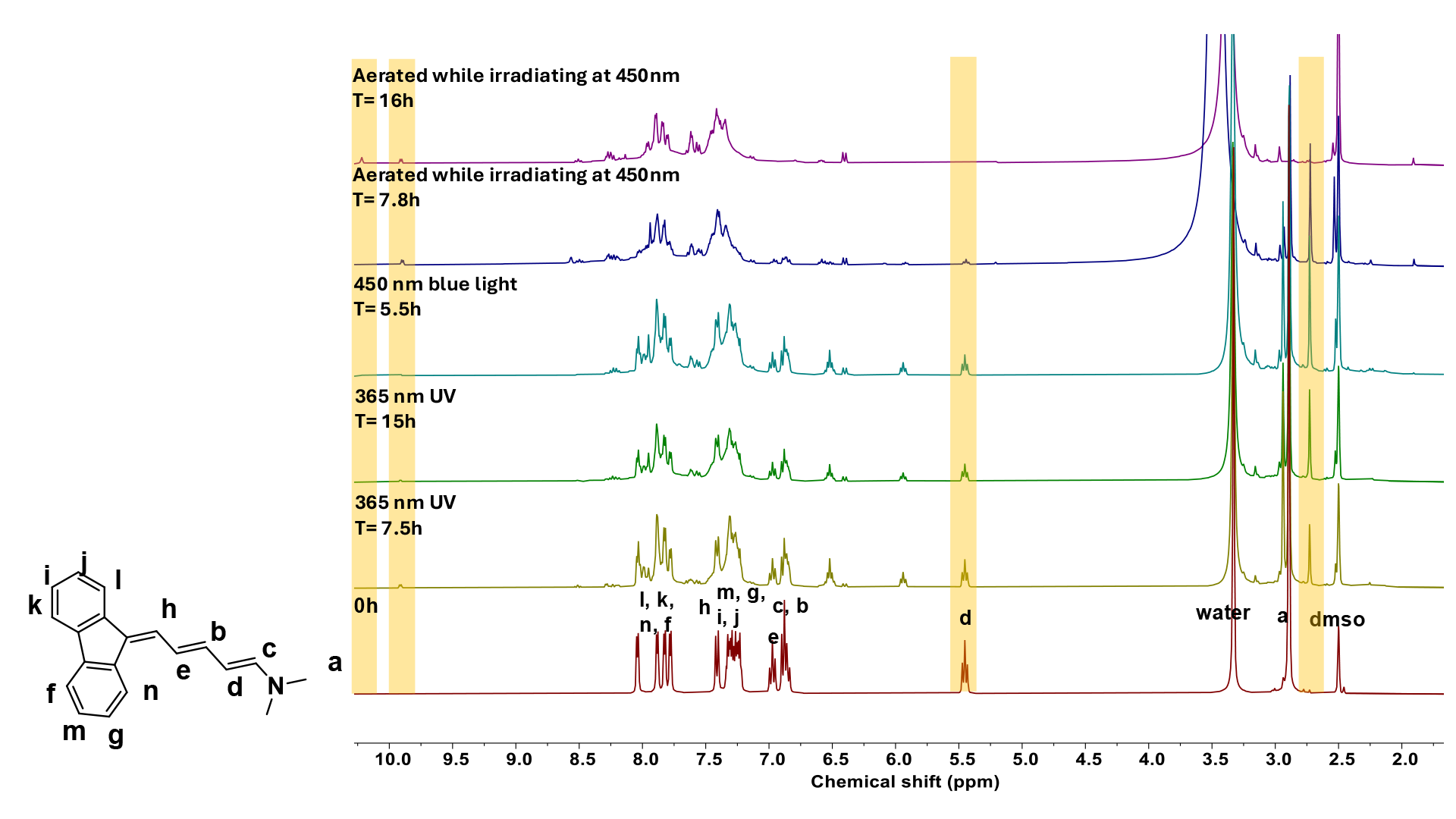


**Figure S9. ^1^H NMR spectra of DB33 over 31 hours of irradiation, incubated at 21 °C (600 MHz, DMSO-*d_6_*).**Irradiation was initially performed for a total of 15h under 365nm UV light. As this did not result in complete photoconversion, the wavelength was increased to 450nm and the samples were exposed to this UV wavelength and aerated for an additional 16h (total of 31h).


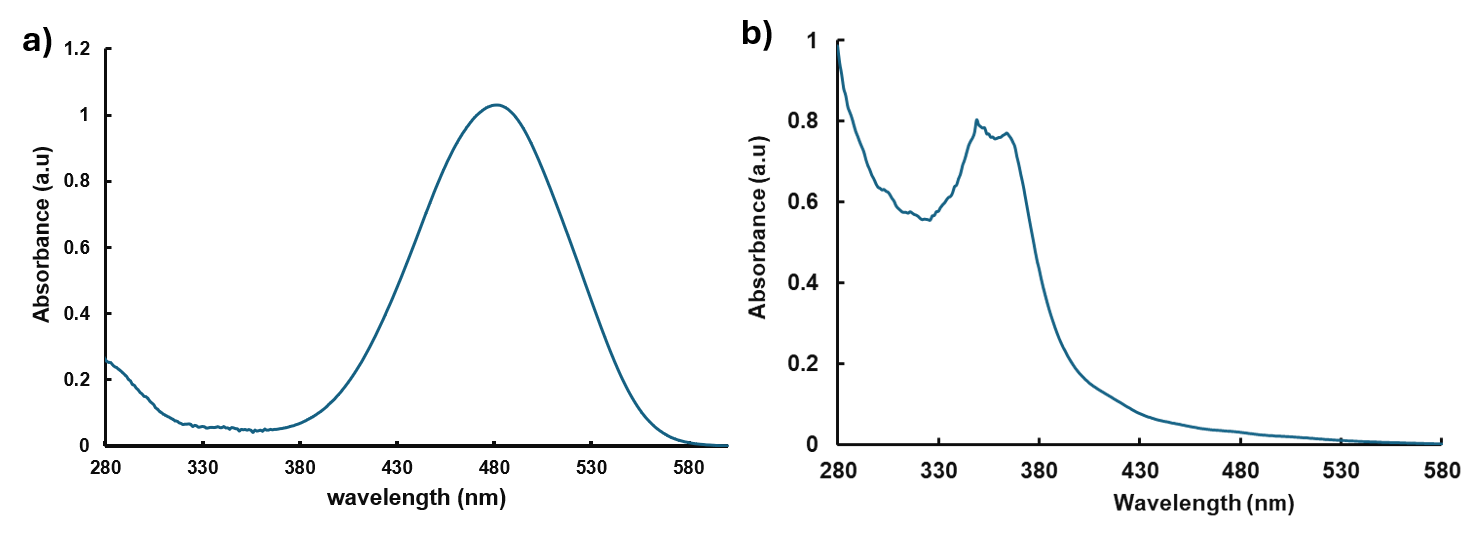


**Figure S10. UVA spectra of DB33.** (A) UV-visible spectrum of DB33 prior to irradiation. (B) UV-visible spectrum of the photochemical degradation products of DB33 after a total of 31 hours of irradiation.

**Figure S11. Mass spectrum of peak *a* from the HPLC separation in Figure 6A.** (A) FTMS + p ESI Full ms [190.0000-600.0000]. HPLC was performed in 1:2:2 DMSO:ACN:H₂O + formic acid, gradient to 95% ACN over 22 min. Peak 235.0002 corresponds to an ACN adduct with the species at 193.9736, however, we were not able to conclusively assign a structure. (B) Re-injection of peak *a* from HPLC in Figure 6A.

**Figure S12. DB33-R breaks down into a highly oxidized intermediate.** (A) HPLC of DB33-Y in 25:75 ACN:H₂O to 100% water over 30 min. FTMS + p ESI Full ms [190.0000-700.0000] of major peaks 5 (B) and 8 (C) from the HPLC in A. Species at 337.1040-1044 is predicted to be the highly oxidized intermediate shown with the formula C_18_H_18_O_5_ (337.1046 g/mol). The presence of multiple peaks in the HPLC trace with the same major mass is consistent with this assignment, as the proposed oxidized structure contains multiple chiral centers and could therefore exist as different stereoisomers that are separable by standard HPLC while producing the same mass spectrum.

**
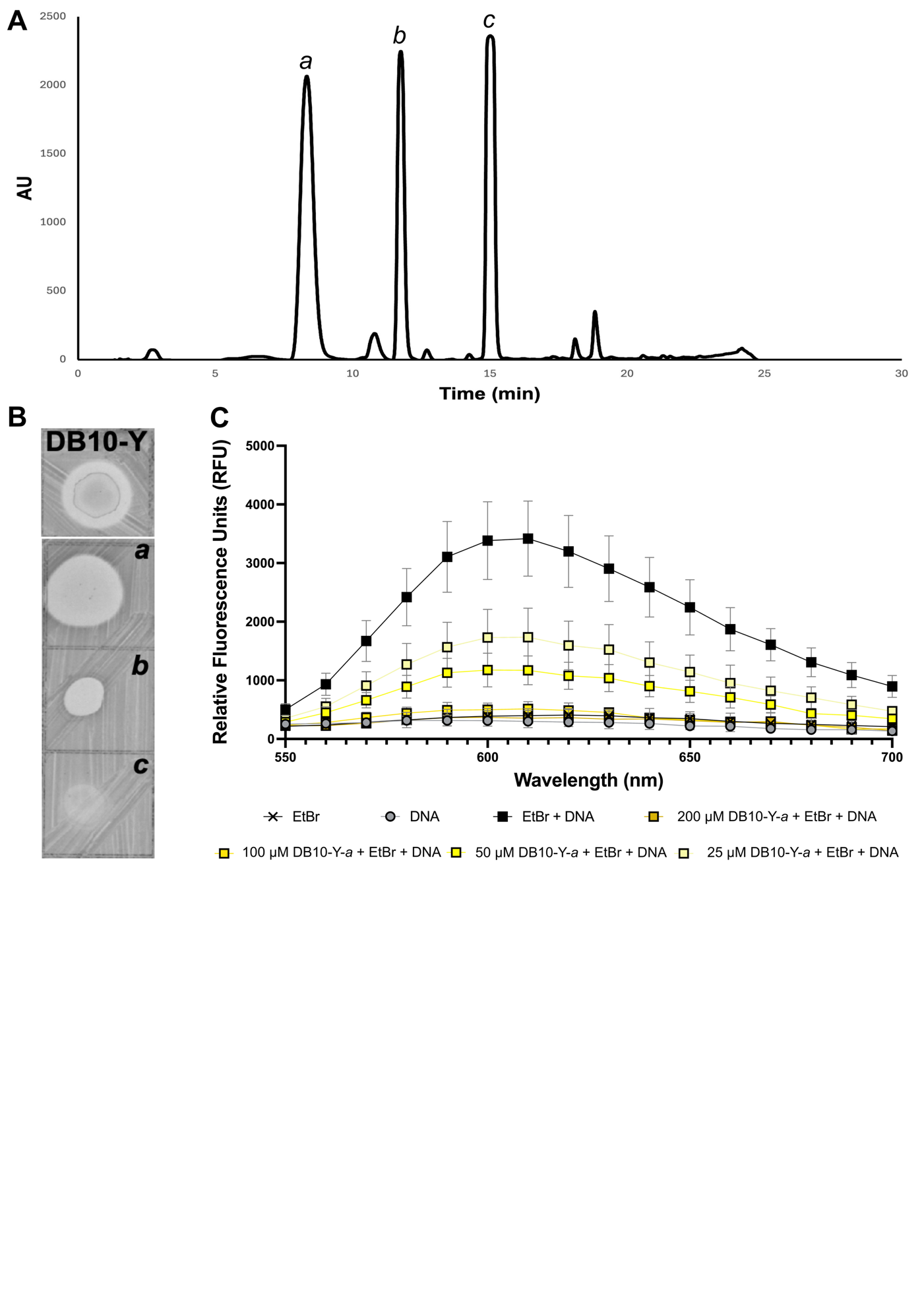
**

**Figure S13. DB10-R breakdown product *a* is bioactive.** (**A**) HPLC trace of the photodegradation products of DB10-R. (**B**) Peaks identified in (A) were collected, dried, and resuspended in DMSO. A 1:1 molar conversion from starting DB10-Y to each breakdown product was assumed to calculate resuspension volumes corresponding to the original DB10-Y stock concentration. Aliquots (3 µL) of each collected peak and the DB10-Y stock were spotted onto MHA plates pre-streaked with WT USA300 LAC. Zones of inhibition were imaged after 24 h incubation. (**C**) DB10-Y-a displaces EtBr from gDNA. Data are shown as the mean ± SD from at least three biological replicates

**Figure S14. DB33-Y inhibits intracellular *S. aureus* replication at non-cytotoxic concentrations and alters the bacterial secretome.** (**A**) Cytotoxicity of DB33 at 200 μM in RAW264.7 macrophages after 24h of exposure. Cytotoxicity was quantified using the LDH release kit and used according to the manufacturer’s instructions. Data are shown as the mean ± SD of at least three independent experiments. ∗p ≤ 0.05, ∗∗p ≤ 0.01, ∗∗∗p ≤ 0.001 using a one-way ANOVA with Dunnett’s multiple comparison. (**B**) Full coomassie brilliant Blue R-250 stained gel of the cropped gel depicted in Figure 7F.

| **Bacterial isolate** | **DB33-R (μM)** | **DB33-Y (μM)** |
| --- | --- | --- |
| *S. aureus* USA100 | 25 | 100 |
| *S. aureus* USA200 | 25 | 100 |
| *S. aureus* USA300 | 25 | 100 |
| *S. aureus* USA400 | 6.25 | 100 |
| *S. aureus* USA600 | 25 | 100 |
| *S. capitis* | 25 | 100 |
| *S. epidermidis* | 25 | 100 |
| *S. chromogenes* | 25 | 100 |
| *S. lugdunensis* | 25 | 100 |
| *S. cohnii* | 50 | 200 |
| *S. warneri* | >200 | >200 |
| *B. subtilis* | 50 | 100 |
| *S. pyogenes* | 12.5 | 25 |
| *S. agalactiae* | 6.25 | 100 |
| *E. faecalis* | 25 | 200 |
| *P. aeruginosa* | >200 | >200 |
| *E. coli* | >200 | >200 |

**Table S1**. **DB33-R and DB33-Y are active against a variety of bacterial species.** MICs of DB33-R and DB33-Y against the same panel of bacterial strains and species previously tested for DB10-R and DB10-Y.

**Table S2. Bacterial strains used in this study**

| **Strain or Plasmid** | **Description** | **Source or reference** |
| --- | --- | --- |
| ***S. aureus*** | | |
| USA300 | USA300 LAC, cured of resistance plasmids | Lab stock |
| RN4220 | r_K_^−^ m_K_^+^; capable of accepting foreign DNA | Lab stock |
| USA100 | WT *S. aureus* USA100 strain | Lab stock |
| BK21203 | WT *S. aureus* USA200 strain, MN8 lineage | Lab stock |
| MW2 | WT *S. aureus* USA400 strain | Lab stock |
| USA600 | WT *S. aureus* USA600 strain | Lab stock |
| USA300 *recA*::tn | WT *S. aureus* USA300 with a transposon mutation of *recA* | NTML library |
| USA300 *rexA*::tn | WT *S. aureus* USA300 with a transposon mutation of *rexA* | NTML library |
| USA300 *rexB*::tn | WT *S. aureus* USA300 with a transposon mutation of *rexB* | NTML library |
| USA300 *clpB*::tn | WT *S. aureus* USA300 with a transposon mutation of *clpB* | NTML library |
| USA300 *clpC*::tn | WT *S. aureus* USA300 with a transposon mutation of *clpC* | NTML library |
| USA300 *clpP*::tn | WT *S. aureus* USA300 with a transposon mutation of *clpP* | NTML library |
| USA300 *clpL*::tn | WT *S. aureus* USA300 with a transposon mutation of *clpL* | NTML library |
| USA300 p*clpX_YR-1_* | WT *S. aureus* USA300 carrying the mutant *clpX* gene (Codon 400 SNP) from USA300 on plasmid pALC2073 | This study |
| USA300 p*clpX_YR-2_* | WT *S. aureus* USA300 carrying the mutant *clpX* gene (-12delC) gene from USA300 on plasmid pALC2073 | This study |
| **Other Staphylococcal species** | | |
| *S. epidermidis* Mach 1457 | Competent clinical strain | Lab stock |
| *S. lugdunensis* M23590 (HM-141) | Human skin isolate | ATCC type strain |
| *S. chromogenes* ATCC 43764 | From the original ATCC stock culture, this isolate possesses a white, mucoid colony | ATCC type strain |
| *S. capitis* ATCC 35661 | Human skin isolate | ATCC type strain |
| *S. warneri* ATCC 27836 | Human skin isolate | ATCC type strain |
| *S. cohnii* ATCC 29973 | Human skin isolate | ATCC type strain |
| **Other Gram-positive bacteria** | | |
| *M. luteus* ATCC 4698 | Human nasal secretion isolate | ATCC type strain |
| *B. subtilis* 3A1T | Wild-type isolate | Bacillus Genetic Stock Center |
| *S. pyogenes* MGAS8232 | Isolated from a patient with acute rheumatic fever. | J. McCormick |
| *S. agalactiae* A909 | Isolated from a septic human neonate | ATCC type strain |
| *E. faecalis* ATCC 33186 | Strain CN478; historical urine isolate | ATCC type strain |
| **Gram-negative bacteria** |  |  |
| *P. aeruginosa* PAO1 | Wildtype strain | K. Poole |
| *E. coli* DH5α | F^−^ ϕ80d*lacZ*ΔM15 *recA1 endA1 gyrA96 thi-1 hsdR17*(r_K_^−^ m_K_^−^) *supE44 relA1 deoR* Δ(*lacZYA-argF*)*U169 phoA* | Promega |
| **Plasmids** | | |
| pALC2073 | *E. coli - S. aureus* shuttle vector. Amp^R^ in *E. coli*, Cm^R^ in *S. aureus* | *82* |

**Table S3. Oligonucleotides used in this study**

| **Name** | **Sequence** | **Description** |
| --- | --- | --- |
| *clpX*-F | TATATA GGTACC ATCTGCTACTATTCTTTAAGC | Forward primer to amplify *clpX* operon from *S. aureus* USA300 LAC |
| *clpX*-R | TATATA GAGCTC GATTGGAGCTTTTCACTTT | Reverse primer to amplify *clpX* operon from *S. aureus* USA300 LAC |

**References**

1. Maoka, T. Carotenoids: Distribution, Function in Nature, and Analysis Using LC-Photodiode Array Detector (DAD)-MS and MS/MS System. *Mass Spectrom (Tokyo)* **12**, A0133 (2023).

2. Informatics, N. O. of D. and. β Carotene.

https://webbook.nist.gov/cgi/cbook.cgi?ID=C7235407&Mask=200#Refs.
